# The Flavone Luteolin Suppresses SREBP-2 Expression and Post-Translational Activation in Hepatic Cells

**DOI:** 10.1371/journal.pone.0135637

**Published:** 2015-08-24

**Authors:** Tsz Yan Wong, Shu-mei Lin, Lai K. Leung

**Affiliations:** 1 Food and Nutritional Sciences Programme, School of Life Sciences, Faculty of Science, The Chinese University of Hong Kong, Shatin, Hong Kong S.A.R., People's Republic of China; 2 Dept. of Food Science, National Chiayi University, Chiayi City, Taiwan, (R.O.C.); 3 Biochemistry Programme, School of Life Sciences, Faculty of Science, The Chinese University of Hong Kong, Shatin, Hong Kong S.A.R., People's Republic of China; Univeristy of California Riverside, UNITED STATES

## Abstract

High blood cholesterol has been associated with cardiovascular diseases. The enzyme HMG CoA reductase (HMGCR) is responsible for cholesterol synthesis, and inhibitors of this enzyme (statins) have been used clinically to control blood cholesterol. Sterol regulatory element binding protein (SREBP) -2 is a key transcription factor in cholesterol metabolism, and *HMGCR* is a target gene of SREBP-2. Attenuating SREBP-2 activity could potentially minimize the expression of *HMGCR*. Luteolin is a flavone that is commonly detected in plant foods. In the present study, Luteolin suppressed the expression of SREBP-2 at concentrations as low as 1 μM in the hepatic cell lines WRL and HepG2. This flavone also prevented the nuclear translocation of SREBP-2. Post-translational processing of SREBP-2 protein was required for nuclear translocation. Luteolin partially blocked this activation route through increased AMP kinase (AMPK) activation. At the transcriptional level, the mRNA and protein expression of SREBP-2 were reduced through luteolin. A reporter gene assay also verified that the transcription of *SREBF2* was weakened in response to this flavone. The reduced expression and protein processing of SREBP-2 resulted in decreased nuclear translocation. Thus, the transcription of *HMGCR* was also decreased after luteolin treatment. In summary, the results of the present study showed that luteolin modulates *HMGCR* transcription by decreasing the expression and nuclear translocation of SREBP-2.

## Introduction

Cardiovascular disease (CVD) is one of the leading causes of morbidity and mortality worldwide. Serum cholesterol levels are correlated with the risk of CVD. A recent meta-analysis estimated that a decrease of 10 mg/dl plasma cholesterol could reduce the mortality of coronary heart disease by 9% in the elderly [1]. Cholesterol homeostasis is tightly controlled in humans through the sterol-regulatory element binding protein (SREBP). SREBP-2 regulates HMG-CoA reductase (HMGCR) expression, which catalyzes the rate-limiting step of cholesterol biosynthesis. HMGCR inhibitors have been prescribed clinically for the treatment of patients with hypercholesterolemia. Thus, influencing HMGCR activity through SREBP-2 could be an alternative approach for treating this disease.

Sterol regulatory element-binding proteins (SREBPs) are basic helix-loop-helix-leucine zipper (bHLH-Zip) family transcriptional factors that regulate lipid metabolism [2]. Three subtypes – *1a*, *1c*, and *2* – have been identified in this membrane-bound transcriptional factor family. The type 1c isoform is involved in fatty acid and glucose metabolism, whereas the type 2 isoform primarily regulates cholesterol biosynthesis. Although the 1a isoform controls all SREBP responsive genes, this transcription factor is not predominantly expressed in the liver.

Under normal physiological conditions, SREBP-2 regulates cholesterol homeostasis through related target genes [3]. When SREBP-2 is ectopically overexpressed, this protein enhances the expression of 12 enzymes that are involved in cholesterol biosynthesis [4], and *HMGCR* is a prime target of SREBP-2 [5]. The rate of cholesterol biosynthesis increased by approximately 28-fold in transgenic mice overexpressing SREBP-2 [2].

The *SREBF2* gene encodes the precursor form (125 kDa) of SREBP-2, and activation occurs through SREBP-cleavage activating protein (SCAP) in a post-translational modification, which is consistent with other SREBP family members. In sterol deficiency, SCAP interacts with SREBP-2 and binds to the coatamer protein II (COPII) vesicle. This complex subsequently migrates from the ER to the Golgi. Site-1 protease (S1P) and Site-2 protease (S2P) in the Golgi sequentially cut the SREBP-2 precursor to release the active transcriptional factor. The cleaved SREBP-2 (approximately 68 kDa) subsequently translocates to the nucleus and binds to Sterol Responsive Element (SRE) target genes. Under high sterol conditions, cholesterol binds to the sterol-sensing domain of SCAP. SCAP undergoes conformational changes and binds to insulin-induced proteins (INSIG-1,-2) instead of SREBP, thereby reducing the nuclear translocation of SREBP-2 [2, 6, 7].

SREBP-2 can be regulated at transcriptional and post-translational levels, and this regulation might involve certain signal transduction pathways. The activation of phosphatidylinositol 3-kinase and Akt facilitates the transport of SREBP-2 to the Golgi for processing. Insulin-activated ERK-1/2 directly phosphorylates SREBP-2 and potentiates the transactivation of this transcription factor [8]. In contrast, AMPK phosphorylates the precursor form of SREBP-2, preventing processing into the active form [9]. In addition, nuclear-bound SREBP-2 undergoes ubiquitination and degradation in the cytosolic 26S proteasome. SREBP-2 ubiquitination occurs independent of cholesterol status, while GSK3-mediated SREBP phosphorylation promotes degradation [10].

Dietary flavonoids are a group of plant pigments with a phenylchoromane or flavone ring [11]. The benefit of flavonoids on hypercholesterolemia and CVD has been demonstrated in many studies. A cross-sectional study on Japanese women demonstrated that increased flavonoid intake is associated with reduced plasma total cholesterol and LDL concentrations [12]. Previous meta-analyses have also shown that isoflavone intake is inversely correlated with plasma LDL cholesterol and triglycerides [13–15].

Luteolin or 3’,4’,5’,7’-tetrahydroxyflavone is a phytocompound isolated from common plant foods. Vegetables, such as celery, broccoli, carrots, thyme, and green peppers, are good sources of this flavonoid. Luteolin is one of the most potent aromatase inhibitors in the flavonoid family *in vitro* [16, 17]. Furthermore, this flavonoid inhibits the transcriptional or enzymatic activity of aromatase in cells [18] and athymic mice [19].

It has been suggested that the fiber content of fruit and vegetables is responsible for the plasma cholesterol-lowering effects of these foods. However, in the present study, we hypothesized that SREBP-2 mediates reductions in cholesterol synthesis that are induced through flavonoids isolated from fruits and vegetables.

## Materials and Methods

### Chemicals

All phytochemicals (baicalein, Cat# 465119 (>98%); flavone, Cat# F2003 (>99%); genistein, Cat# G6776 (~98%); α-naphthoflavone, Cat# N5757 (>98%); luteolin, Cat# L9283 (>98%); naringenin, Cat# N5893 (>95%); quercetin, Cat# Q0125 (>98%); resveratrol, Cat# R5010 (>99%); chrysin, Cat# C80105 (>97%); hesperetin, Cat# W431300 (>95%); and isoliquiritigenin, Cat# I3766 (>98%)) were obtained from Sigma Chemical (St Louis, MO, USA). The impurities of the phytochemicals could be a confounding factor. Kinase inhibitors, including SB203580 (Cat# 559389, Merck), H-89 (Cat# 371963, Merck), Compound C (Cat# 171260, Merck), Bisindolylmaleimide I (Cat# 203290, Merck), pAKT inhibitor (Cat# 124011, Merck) and U0126 (Cat# 662005, Merck), were purchased from Calbiochem (San Diego, CA, USA). LY333531 (Cat# sc-364215) and HBDDE (Cat# sc-202174) were obtained from Santa Cruz Biotechnology (Santa Cruz, CA, USA). SP600125 (Cat# S5567) and all other chemicals, if not stated, were acquired from Sigma Chemicals (St Louis, MO, USA).

### Cell culture

Liver cancer HepG2 cells and non-cancer WRL cells (American Type Culture Collection, Rockville, VA, USA) were cultured in RPMI– 1640 phenol red-free media (Sigma Chemicals) supplemented with 10% fetal bovine serum (Invitrogen Life Technology, Rockville, MD) and incubated at 37°C and 5% carbon dioxide. These cells were routinely subcultured at 80% confluency. Three days prior to the experiment, the cultures were switched to RPMI– 1640 phenol red-free media (Sigma Chemicals) containing 5% charcoal-dextran-treated fetal bovine serum (Hyclone, Utah, USA). Sub-confluent cell cultures were treated with various concentrations of luteolin with DMSO as the carrier solvent. The final concentration of the solvent was 0.1% v/v, and control cultures received DMSO only. The cell density in each experiment was maintained at 5 × 10^2^ cells/mm^2^.

### Quantitative Real Time RT-PCR assay

Hepatic cells were seeded onto 6-well Costar plates and subjected to various treatments. After 24 h, total RNA was extracted from the cells using TRIzol reagent (Invitrogen, Carlsbad CA, USA). The RNA concentration and purity were determined based on the absorbance measured at 260/280 nm. First-strand DNA was synthesized from 3 μg of total RNA using oligo-dT primers and M-MLV Reverse Transcriptase (USB Corporation, Cleveland, Ohio, USA). Target fragments were quantified through real-time PCR using an ABI prism 7700 Sequence Detection System (Applied Biosystems). Taqman/VIC MGB probes and primers for *SREBF2* (Cat# 4331182-HS01081784_M1), *HMGCR* (Cat# 4331182-HS00168352_M1), *LDLR* (Cat# 4331182- HS00181192_M1) and *GAPDH* (Cat# 4326317E) (Assay-on-Demand) as well as the Real-time PCR Taqman Universal PCR Master Mix were all obtained from Applied Biosystems. PCR reactions were prepared according to the manufacturer’s instructions. The signals obtained for GAPDH served as a reference to normalize the amount of RNA amplified in each reaction. Relative gene expression was analyzed using the 2^‒∆∆CT^ method [20].

### Luciferase reporter gene assay

A fragment from the 5’-region flanking *HMGCR* or *SREBF2* was amplified from human genomic DNA using the primers shown in Table 1. The polymerase chain reaction (PCR) product was digested with KpnI and XhoI and subcloned into the firefly luciferase reporter vector pGL3 (Clontech, Palo Alto, CA, USA).

**Table 1:** Primer sequences for reporter plasmid construction.

	Oligonucleotide	Sequence
*HMGCR*	-1194 Forward	CGGGGTACCACCCTCCCTTTCTACCTTGTG
	-49 Reverse	CCGCTCGAGACTTTCCTGTGCGAACCTTAC
*SREBF2*	-772 Forward	CGGGGTACCGTGAGGTGCTTGAAGGAGTGGG
	-96 Reverse	CCGCTCGAGAGCCAATGGGCGAGCGAAG

GGTACC and CTCGAG are the respective restriction sites for KpnI and XhoI.

WRL-68 cells were seeded onto 96-well plates. After 24 h, the cells were transiently transfected with 0.25 μg of the *HMGCR* promoter-driven firefly luciferase reporter plasmid and 3.0 ng of Renilla luciferase control vector (Promega, Madison, WI, USA) in Lipofectamine (Invitrogen Life Technologies). After 6 h, the medium was removed, and the cells were treated with various concentrations of luteolin for 24 h. The cells were lysed, and the luciferase substrates (provided in the Dual-Luciferase Assay Kit, Promega) were mixed with the cell lysate. Luciferase bioluminescence was measured using a FLUOstar Galaxy plate reader according to the manufacturer’s instructions. The *HMGCR* transactivation activity, represented as firefly luciferase light units, was normalized to that of Renilla luciferase.

### Electrophoretic mobility shift assay

The nuclear protein extract was isolated using a NucBuster protein extraction kit (Novagen, EMD Biosciences, Inc., La Jolla, CA, USA). Briefly, the cells were washed, trypsinized, and centrifuged at 500 × g at 4°C. Reagent 1 was added to the packed cells. Nuclear extract was isolated from the cell suspension through vortexing and centrifugation. The nuclear protein was stored at -80°C until further use. An oligonucleotide mimicking (-160 to -141) *HMGCR* (Table 2) was synthesized and labeled using the DIG Gel Shift Kit, 2^nd^ Generation (Roche Diagnostics GmbH).

**Table 2:** Oligonucleotide sequences for Electrophoretic Mobility Shift Assay.

***HMGCR* (-160 to -141)**	5’-GTT GGC CGA GCC CGT GGT GAg aga tgG TGC GGT Gcc tgt tct tgg -3’
**7×SRE**:	5'-gtg cgg tgg tgc ggt ggt gcg gtg gtg cgg tgg tgc ggt ggt gcg gtg gtg cgg tg-3'

The underlined sequences are SRE binding motifs.

The nuclear protein was incubated with the labeled probe, sonicated salmon sperm DNA, poly(dI-dC), and binding buffer (400 mm KCl, 80 mm HEPES, 2 mm DTT, 0.8 mM EDTA, pH 8 and 80% glycerol) provided in the Electrophoretic Mobility Shift Assay Accessory Kit (Novagen) for 30 min at room temperature. The 7×SRE (Table 2) unlabeled oligonucleotide or SREBP-2 antibody was co-incubated as the competitive control. The reaction mix was subsequently separated on a 4–6% non-denaturing gel in 0.5 × Tris-borate EDTA at 100 V. The labeled oligonucleotide-protein complex was electro-transferred to a nylon membrane, fixed using *UV* light, blocked and washed. The shifted oligonucleotide was detected using anti-Digoxigenin-AP conjugate and the chemiluminescent substrate CSPD provided in the kit.

### Western blot analysis

The cells were washed once with PBS (pH 7.4) and harvested in a 1.5-ml microtube containing 0.5 ml of lysis buffer (PBS, 1% NP40, 0.5% sodium deoxycholate, and 0.1% SDS). The lysis buffer contains protease inhibitors (40 mg/L PMSF, 0.5 mg/L aprotinin, 0.5 mg/L leupeptin, 1.1 mmol/L EDTA and 0.7 mg/L pepstatin) and a phosphatase inhibitor cocktail (PhosphoSTOP tablets, Roche). The harvested cells were subsequently lysed using a cell disruptor (Branson Ultrasonics Corp., Danbury, CT, U.S.A.) on ice for 30 s. The protein concentration of the cell lysate was determined using the DC Protein Assay (BioRad, Richmond, CA, U.S.A.). A total of 50 μg of the protein lysate was on 10% SDS-PAGE and transferred onto an Immobilon PVDF membrane (Millipore, Bedford, MA, U.S.A.). Primary antibodies for SREBP-2 (Cat# sc-13552, Santa Cruz Biotechnology), phospho-ERK-1/2 (Cat# sc-7383, Santa Cruz Biotechnology), phospho-PKA (Cat# sc-21901-R, Santa Cruz Biotechnology), phospho-JNK (Cat# sc-6254, Santa Cruz Biotechnology), N-terminal SREBP-2 (Cat# ab30682, Abcam PLC, Cambridge, UK), HMGCR (Cat# ABS229, Millipore, Bedford, MA), phospho-PKC isoforms (Cat# 9371, 9375, 9376, 9378, 9379, Cell Signaling Technology, Danvers, MA, USA.), t- and p-AMPK (Cat# 2532 and 2535, Cell Signaling Technology), phospho-P38 (Cat# 9221, Cell Signaling Technology, Danvers, MA, USA) and β-actin (Cat# A-5316, Sigma Chemicals) and secondary antibodies conjugated with horseradish peroxidase (anti-rabbit-HRP, Cat# sc-2004, Santa Cruz Biotechnology; anti-mouse-HRP, Cat# 401215, Millipore) were used for protein detection. The chemiluminescence substrate for HRP was obtained from an ECL Detection Kit (Amersham, Arlington Heights, IL, U.S.A.), and the targeted protein was visualized using autochemiluminography.

The NucBuster protein extraction kit (Novagen) was used to prepare the nuclear and cytosolic protein lysates as described above.

### Immunocytochemical imaging

WRL-68 cells were grown on 35-mm glass bottom dishes and treated with 10 μM luteolin at 40–50% confluence for 24 h. After treatment, the cells were fixed with 4% paraformaldehyde in PBS with 0.2% (v/v) Tween 20 for 5 min, followed by blocking in 3% BSA in PBS for 30 min at room temperature. The dishes were washed and incubated with anti-SREBP-2 and anti-golgin-97 primary antibody (1:100 dilution in PBS) for 3 h. Subsequently, the dishes were incubated with Alexa Fluor 488-labeled (Molecular Probes, Eugene, OR, USA) and Alexa Fluor 568-labeled (Molecular Probes, Eugene, OR, USA) secondary antibodies for 1 h. The dishes were stained with 2-(4-amidinophenyl)-1H -indole-6-carboxamidine (DAPI), and the cells were examined through confocal microscopy.

### Transfection of AMPK siRNA

HepG2 cells were cultured in OptiMEM (Invitrogen Life Technology) and transfected with AMPKα1/2 siRNA (sc-45312 Santa Cruz Biotechnology) in Lipofectamine 2000 (Invitrogen Life Technology). At six hours after transfection, the culture medium was replaced with RPMI (phenol red-free) supplemented with 5% charcoal-dextran-treated fetal bovine serum (Biotechnics Research, CA USA), and 25 μM luteolin was subsequently added, followed by incubation for 24 h.

### AMP/ATP assay

The cellular AMP and ATP was extracted using the boiling water method [21]. The cells were seeded onto six-well Costar plates and treated with various concentrations of luteolin for 24 h. The cells were washed twice with cold PBS, followed by the addition of ice-cold water. The cells were scraped into a 1.5-ml tube and lysed using a cell disruptor (Branson Ultrasonics Corporation) on ice for 10 sec. The protein concentration of the cell lysate was determined using a BCA assay (Thermo, South Logan, UT, USA). The remaining lysate was boiled with shaking for 10 min, cooled on ice for 30 s and centrifuged at 13000 rpm for 5 min. The supernatant was collected and stored at -80°C until further use. The levels of ATP, ADP and AMP were determined using an ATP/ADP/AMP Assay Kit (Cat #: A-125; Biomedical Research Service Center, University at Buffalo, State University of New York). The luciferase bioluminescence was measured using a Tecan Infinite M200 luminometer. As described in the protocol, the samples were incubated with or without AMP/ADP-CB/CE reagents (provided in the kit), and the differential readings corresponded to the AMP and ATP concentrations in the samples.

### Cellular cholesterol determination

The intracellular total cholesterol contents in HepG2 cells were measured as previously described [22, 23]. The cells were preincubated overnight in serum-free medium supplemented with 1% BSA. After removing the media, the cells were treated with various concentrations of luteolin for 24 h. The cells were washed with ice-cold PBS and transferred to a 1.5-ml tube. The cells were lysed using a cell disruptor (Branson Ultrasonics Corporation) for 10 s on ice. The protein concentration of the lysate was determined using a BCA assay (Thermo, South Logan, UT, USA). The lipids were extracted using a 2:1 chloroform:methanol (v/v) solvent and centrifuged at 3000 rpm for 10 min. An aliquot of the organic phase was dried in nitrogen. The cholesterol concentration was determined using a commercial enzymatic kit (Stanbio Laboratories, Boerne, TX, USA). The samples were incubated with the kit reagent at 37°C for 5 min, and the formed qunoneimine chromogen was detected based on the absorbance measured at 500 nm. The cholesterol concentration was estimated from a standard curve generated using the cholesterol standard provided in the kit.

### Statistical methods

The Prism^®^5.0 (GraphPad Software, Inc., CA, USA) software package was utilized for statistical analysis. The results were analyzed using *ANOVA* with *Dunnett's post hoc test*, and the significance level was set at *p*<0.05.

## Results

### Effect of flavonoids on *SREBF2* expression in hepatic cells

**Fig 1:**
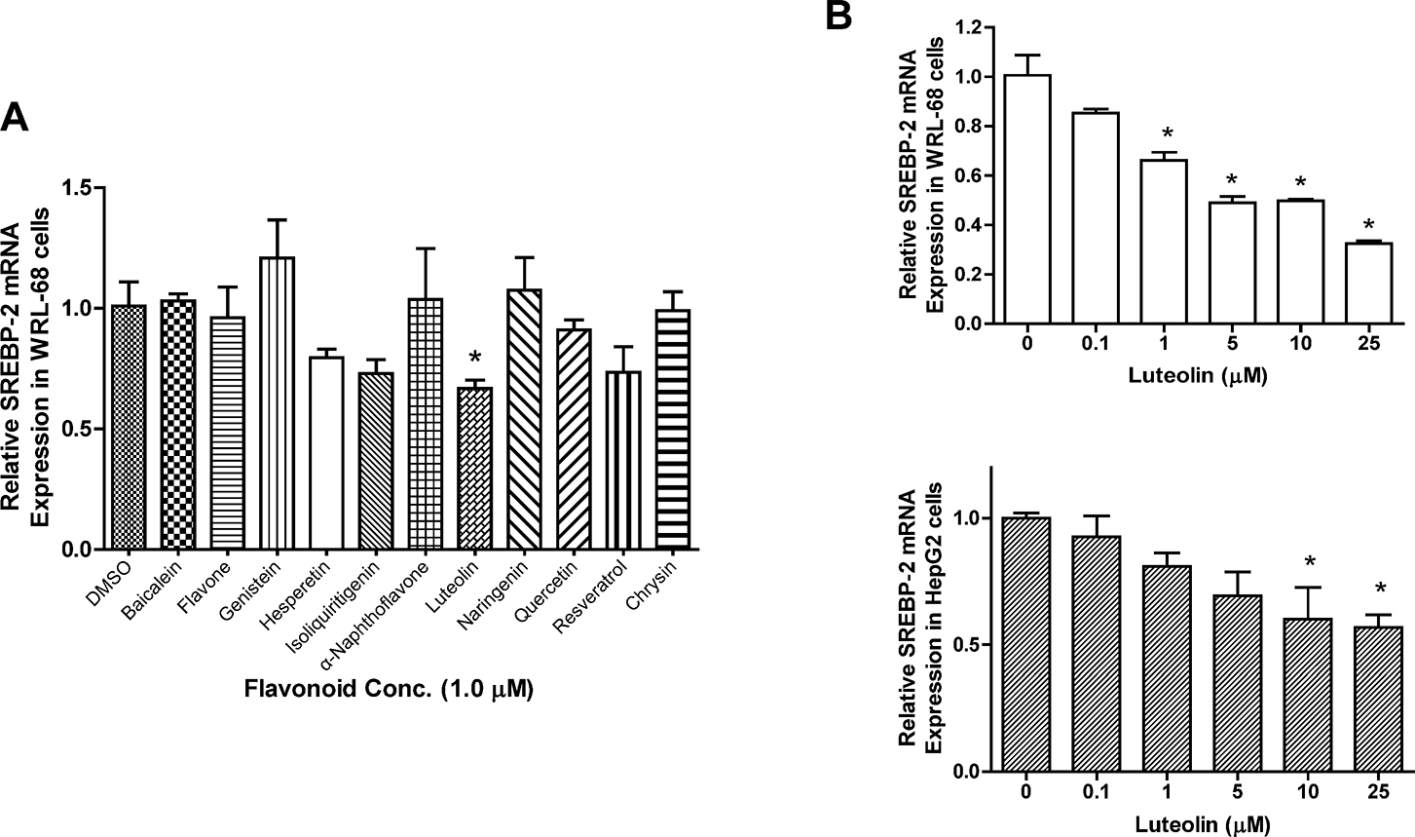
Differential effects of flavonoids on *SREBF2* mRNA expression. The hepatic cells WRL-68 were seeded onto 6-well culture plates and treated with various flavonoids at 1 μM. After 24 h of treatment, total mRNA samples were extracted from the cells. *SREBF2* mRNA expression was determined using real-time RT-PCR (Fig 1A). Dose-response experiments were performed with luteolin at 0, 0.1, 1, 5, 10 and 25 μM in WRL-68 (upper panel) and HepG2 cells (lower panel) as a follow-up to the screening (Fig 1B). The values are presented as the means ±SEM, n = 3 samples per treatment. Means labeled with (*) are significantly different.

*SREBF2* mRNA expression was determined in WRL-68 cells treated with various flavonoids (**Fig 1A**). Given the same treatment concentration at 1 μM for all compounds, luteolin was the most efficacious in impeding the expression of *SREBF2*. The two most commonly investigated compounds, genistein and resveratrol, did not suppress *SREBF2* expression. A dose-response experiment was performed using WRL-68 (**Fig 1B**) and HepG2 (**Fig 1C**) cell cultures treated with luteolin, and a decrease in *SREBF2* expression was observed. The C(t) values used for constructing Fig 1A–1C are shown in **Tables A,B,** and **C** in the **S1 Dataset**.

### Immunoblot of SREBP-2 protein

**Fig 2:**
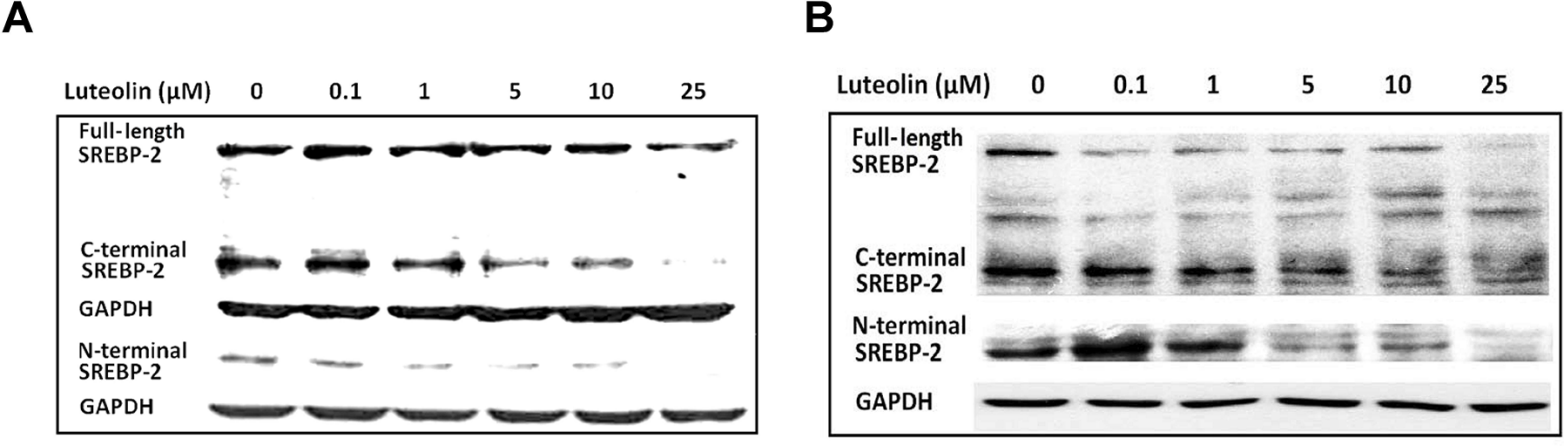
Immunoblot of SREBP-2 under luteolin treatment. Hepatic cells were cultured and treated with luteolin. After 24 h of treatment, the protein extracts were immunoblotted for SREBP-2. Images for SREBP-2 in WRL-68 cells and HepG2 cells are displayed in the left and right panels, respectively. The images represent one of two independent experiments with comparable results.

The precursor form of SREBP-2 was cleaved into C- and N-terminal fragments, and the N-fragment, or N-SREBP-2, represented the active transcriptional factor. Further analysis revealed that reduced N-SREBP-2 was detected after luteolin treatment in WRL-68 (**Fig 2A**) and HepG2 (**Fig 2B**) cells. **Figures A** and **B** in the **S2 Dataset** contain images obtained from the three trials.

### Transcriptional activities of *SREBF2* in luteolin-treated cells

**Fig 3:**
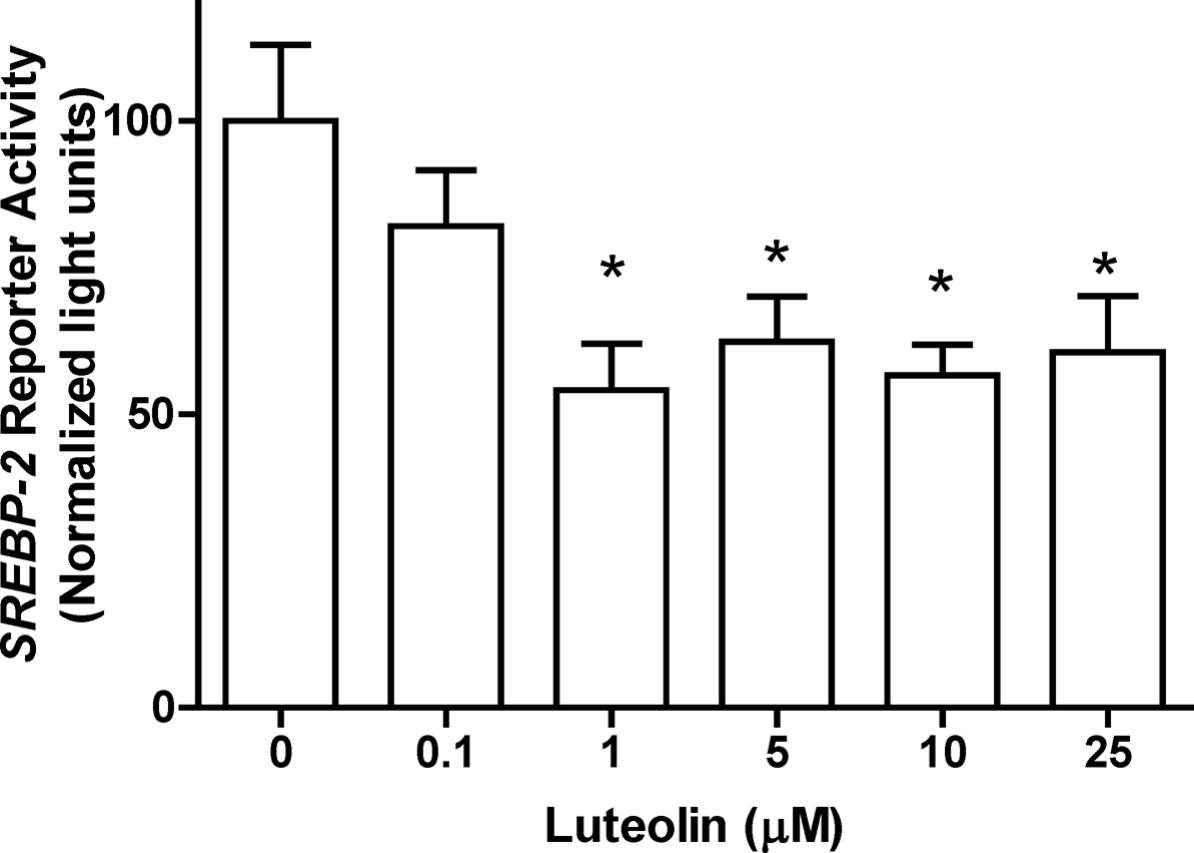
Luteolin suppressed *SREBF2*-driven luciferase activity. WRL-68 cells were transiently transfected with a firefly luciferase reporter gene driven through the *SREBF2* fragment (-772 to -96) and a renilla luciferase control plasmid (pRL). The cells were treated with 0, 0.1, 1, 5, 10, and 25 μM luteolin for 24 h. The values are presented as the means ±SEM, n = 5 samples per treatment. Means labeled with (*) are significantly different.

As luteolin repressed *SREBF2* mRNA expression, the regulation of the *SREBF2* gene was examined using a reporter gene system. The *SREBF2*-driven luciferase activity was significantly repressed through luteolin at 1 μM (**Fig 3**), and supporting information is shown in the **S3 Dataset, Table A**.

### *SREBF2* transcript expression was altered through protein kinase inhibitors

**Fig 4:**
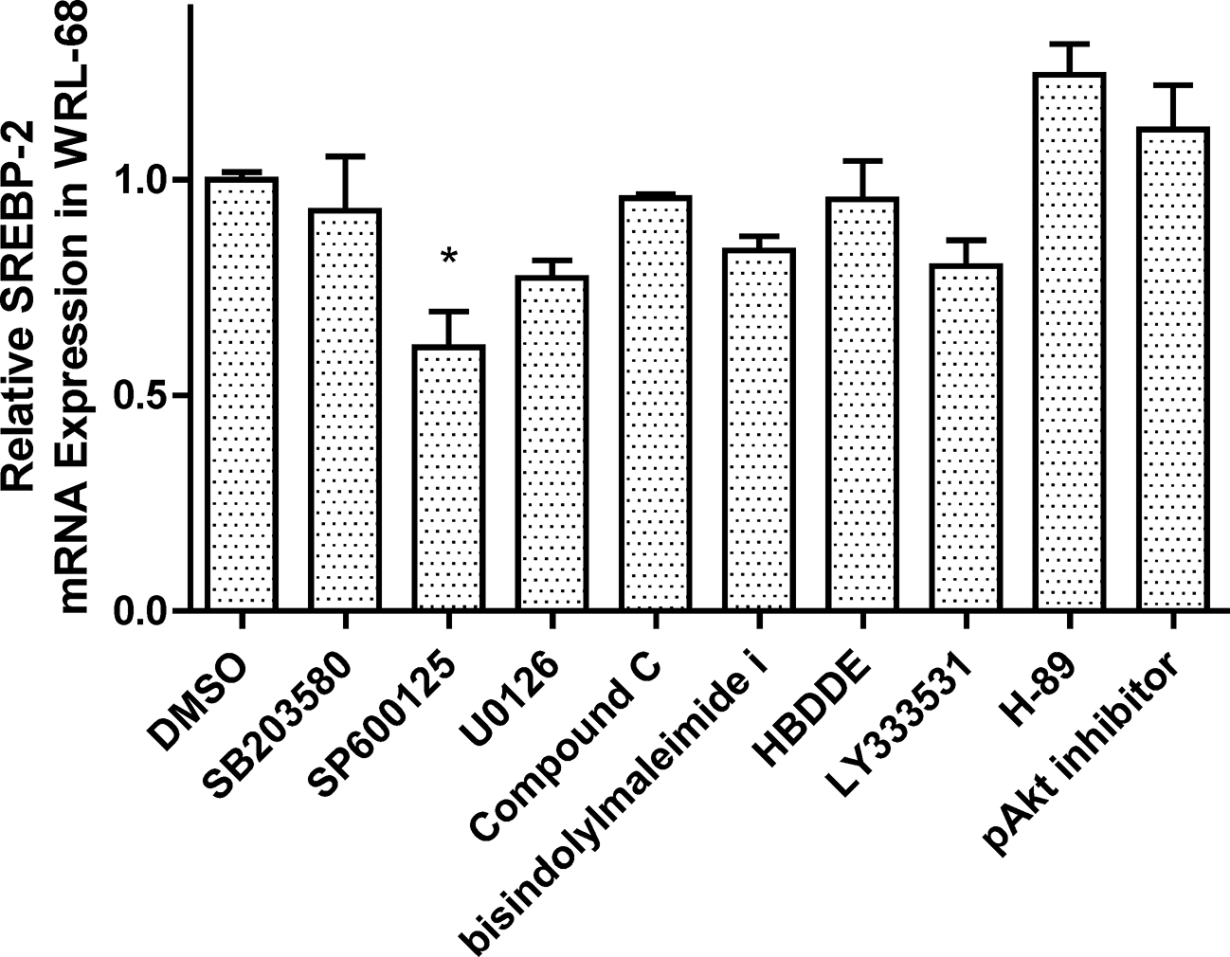
Effect of protein kinase inhibitors on *SREBF2* transcription. WRL-68 were seeded onto 6-well culture plates and pre-treated with various protein kinase inhibitors, including SB203580 (p38), SP600125 (JNK), U0126 (ERK-1/2), Compound C (AMPK), bisindolylmaleimide I (PKCs), HBDDE (PKCα,γ), LY333531 (PKCβ-1/2) and H-89 (PKA). After 24 h of treatment, total mRNA samples were extracted from the cells. *SREBF2* mRNA expression was determined using real-time RT-PCR. The values are presented as the means ±SEM, n = 3 samples per treatment. Means labeled with (*) are significantly different.

As previous studies have shown that the transcription of *SREBF2* is regulated through protein kinases [24, 25], we attempted to identify the potential signal transduction pathways. The JNK inhibitor SP600125 significantly reduced *SREBF2* mRNA expression. Inhibiting other pathways dictated through various protein kinases, such as PKC, PKA, ERK-1/2, AMPK and p38, did not affect *SREBF2* mRNA expression (**Fig 4**), and supporting information is provided in the **S4 Dataset, Table A**.

### The status of protein kinases in hepatic cells treated with luteolin

**Fig 5:**
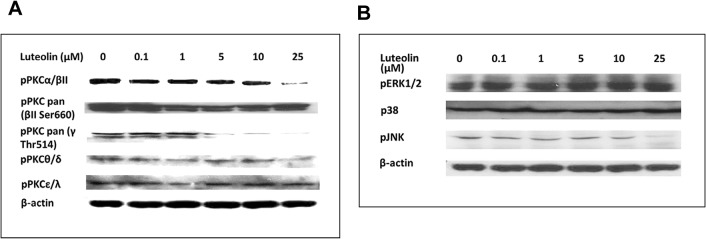
Luteolin attenuated PKCs and MAPKs. WRL-68 cells were cultured and treated with various concentrations of luteolin. After 24 h of treatment, the cell lysates were immunoblotted for Protein Kinase Cs (Fig 5A) and Mitogen Activated Protein Kinases (Fig 5B). The images represent one of two independent experiments with comparable results.

The activation of protein kinases, including PKC isoforms (**Fig 5A**) and MAPKs (**Fig 5B**), in cells treated with luteolin was determined through western blot analysis. The results revealed reductions in p-JNK and p-PKC-α,βІІ and γ; however, PKC inhibition, as depicted above, did not reduce *SREBF2* mRNA levels. JNK was the sole factor attributing to this decrease. **Figures A** and **B** in the **S5 Dataset** show the images obtained from the 3 trials.

### Immunocytochemical staining of SREBP-2 protein

**Fig 6:**
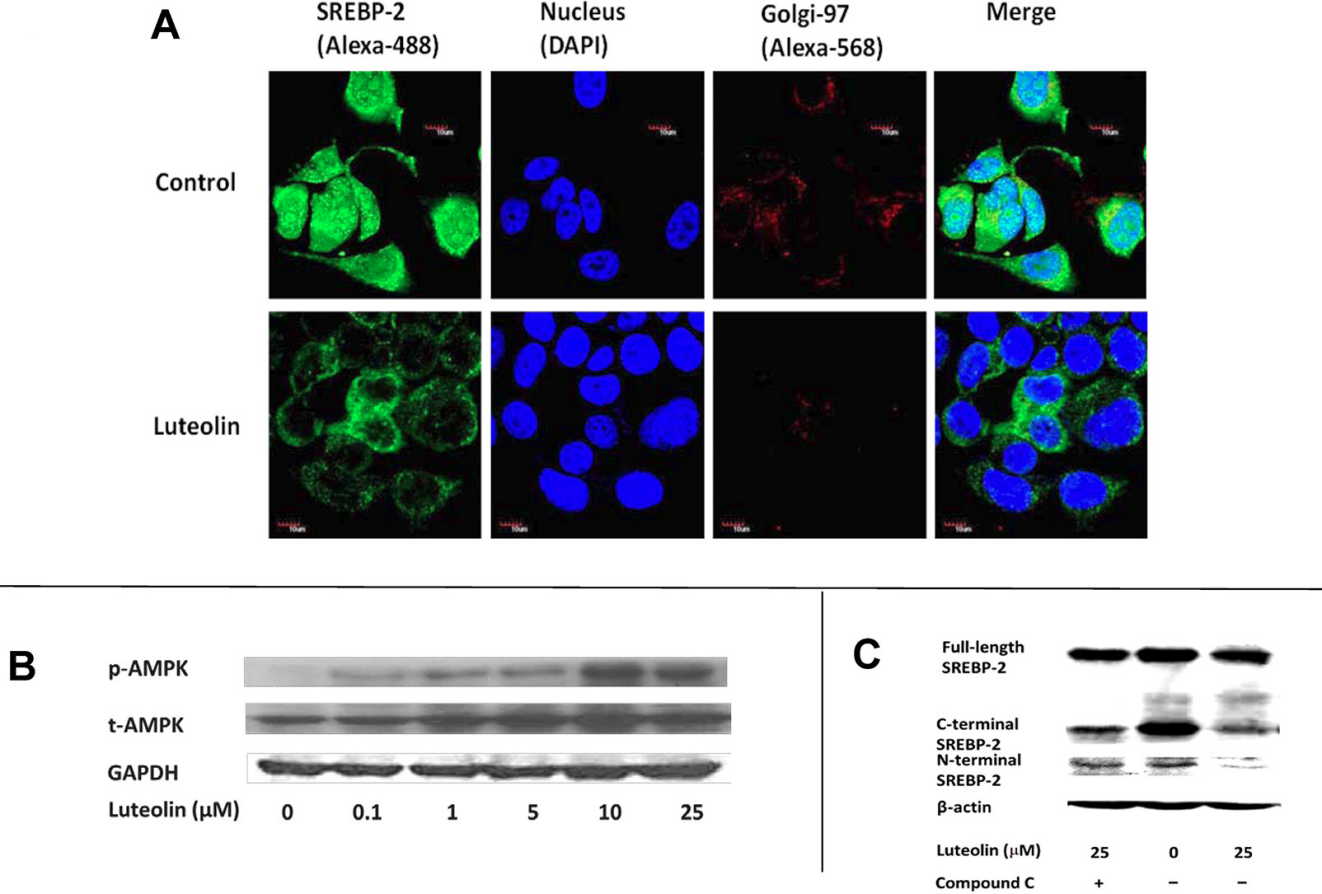
AMPK status and nuclear translocation of SREBP-2 in luteolin-treated WRL-68 cells. The hepatic cells WRL-68 were seeded onto 6-well culture plates and treated with luteolin at 10 μM. After 24 h of treatment, the cells were fixed and incubated with Golgi-specific and SREBP-2 primary antibodies and fluorophore (Alexa 568 and 488)-labeled secondary antibodies. The nuclei were counterstained with DAPI (Fig 6A). The images were obtained using confocal microscopy. In a separate experiment, the cultures were treated with various concentrations of luteolin and co-treated with the AMPK inhibitor Compound C. After 24 h of treatment, the cell lysates were immunoblotted for AMPK and SREBP-2. Images for AMPK and SREBP-2 are displayed in the lower left (Fig 6B) and right panels (Fig 6C), respectively. These results represent one of two independent experiments.

Because the expression of SREBP-2 was reduced, the translocation of the transcription factor should also be decreased correspondingly. Compared with the control, the Alexa-488-labeled SREBP-2 in cells treated with luteolin was low. Compared with the DAPI-labeled nuclei image, the labeled SREBP-2 protein under luteolin treatment was primarily distributed in the cytosol, as shown in the **Merge** image (**Fig 6A**). This uneven distribution indicated that luteolin prevented the translocation of SREBP-2. Images from the 3 trials are shown in the **S6 Dataset, Figure A**.

### Role of AMPK in SREBP-2 processing

As previous studies have shown that protein kinases might participate in the processing and activation of SREBP-2, we examined the status of some protein kinases under luteolin treatment. AMPK is important for the regulation of SREBP-2 processing, and this kinase was activated through luteolin as shown in **Fig 6B**. A follow-up study was conducted to show the effects of luteolin-activated AMPK. The AMPK-specific inhibitor, compound C reversed the luteolin-reduced cleavage of SREBP-2 (**Fig 6C)**. The **S6 Dataset, Figures B** and **C,** display the immunoblot images obtained from the 3 trials. This result illustrated that luteolin-activated AMPK is involved in the decreased processing of SREBP-2 precursor protein.

### SRE-driven luciferase activities and EMSA assay

**Fig 7:**
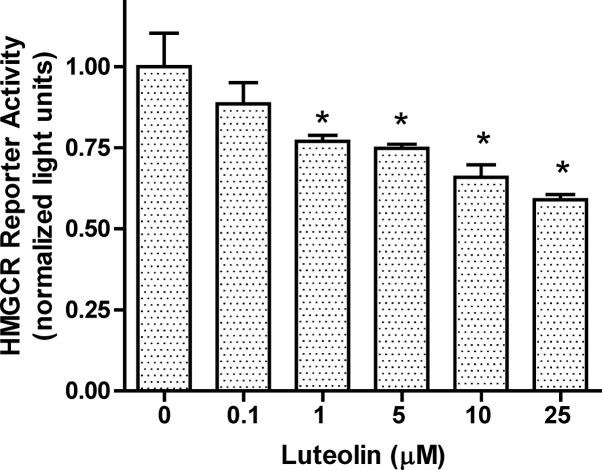
Effect of luteolin on SRE-driven luciferase activity. WRL-68 cells were transiently transfected with a firefly luciferase reporter gene driven by SRE-containing *HMGCR* fragment (-1194 to -49) and a renilla luciferase control plasmid (pRL). The cells were treated with 0, 0.1, 1, 5, 10, and 25 μM luteolin for 24 h. The values are presented as the means ±SEM, n = 3 samples per treatment. Means labeled with (*) are significantly different.

**Fig 8:**
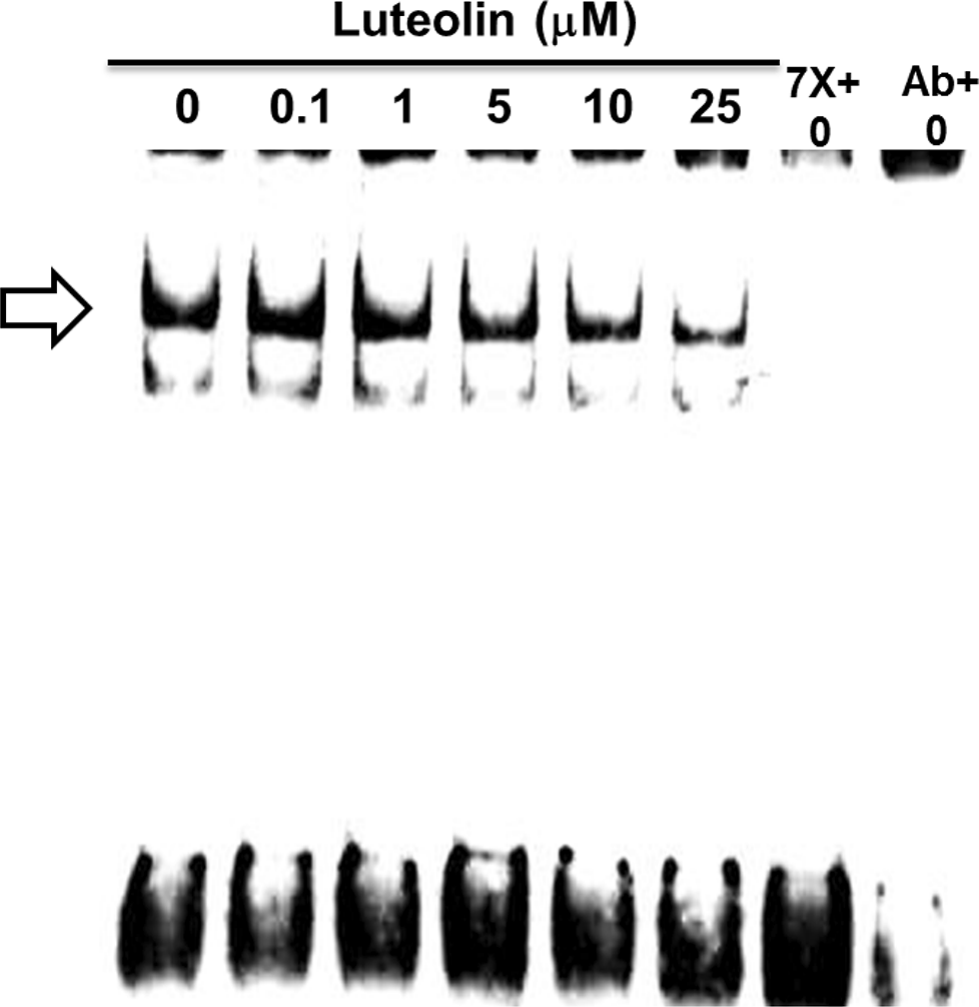
Luteolin weakened the SRE-DNA interaction. The hepatic cells WRL-68 were seeded onto 6-well culture plates and treated with luteolin at 0, 0.1, 1, 5, 10, and 25 μM. After 24 h, nuclear extracts were obtained from the cells and EMSA assay was performed. (⇨) indicates the SREBP-2-SRE interaction band. The image represents one of two independent experiments.

SREBP-2 transactivation represents the most common regulation for *HMGCR* expression. Considering that luteolin interferes with SREBP-2 translocation, the transcriptional regulation of downstream genes was evaluated. The SRE-driven luciferase activity was significantly repressed after treatment with luteolin at 1 μM (**Fig 7 and Table A** in the **S7 Dataset**). The EMSA assay was used to examine the interaction between the N-SREBP-2 and SRE motifs (**Fig 8**) (**Figure A** in the **S8 Dataset**). The position of the interacting band was revealed after co-incubation with the 7× SRE unlabeled oligonucleotide fragment or anti-N-SREBP-2. The band was competed out after either treatment. The data showed that this interaction was decreased in WRL-68 cells after treatment with luteolin.

### Messenger RNA expression of *HMGCR*, *PCSK9* and *LDLR*

**Fig 9:**
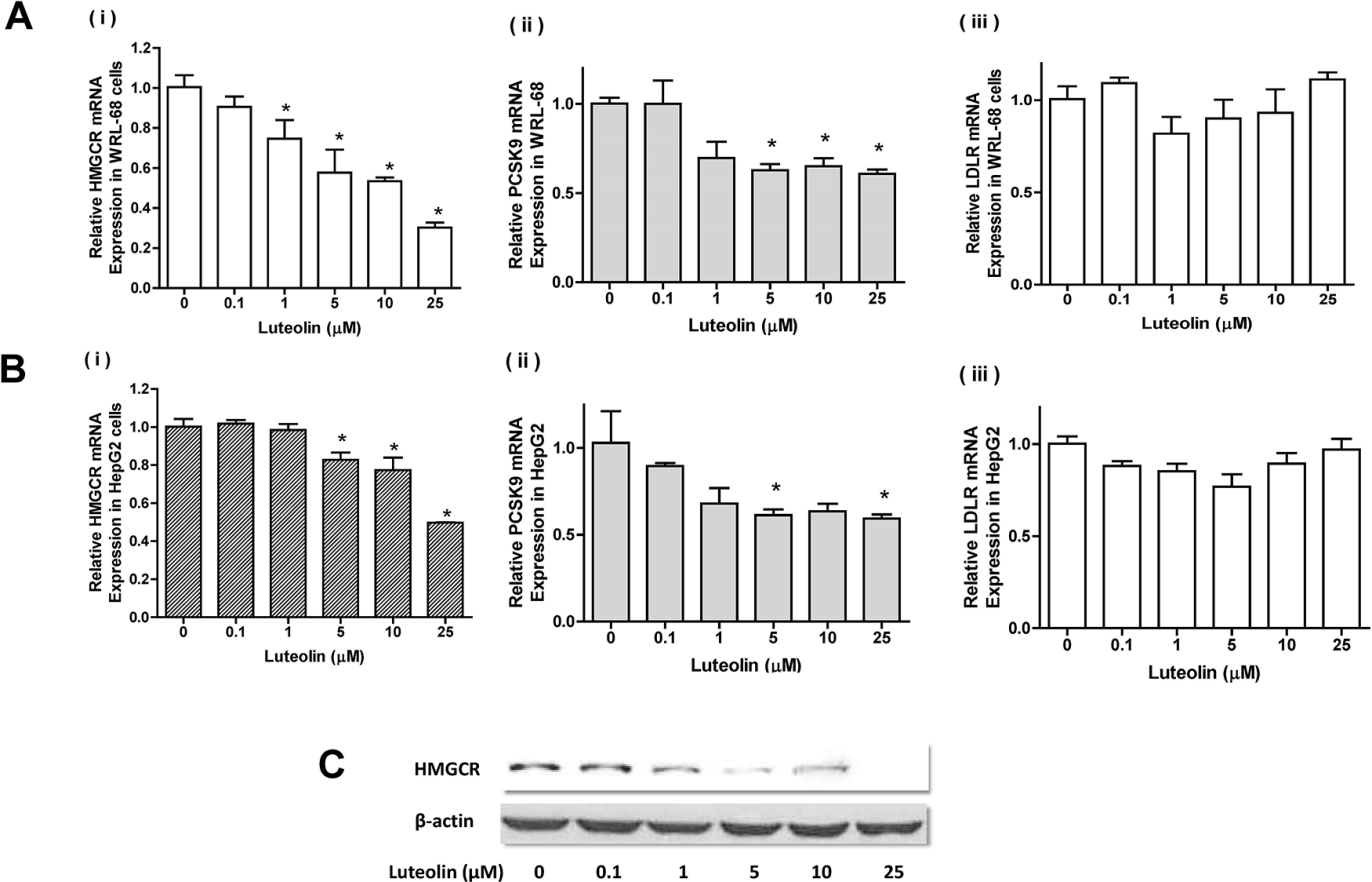
Expression of *HMGCR*, *PCSK9* and *LDLR* in luteolin-treated hepatic cells. WRL-68 and HepG2 cells were treated with various concentrations of luteolin and cultured for 24 h. Messenger RNA of *HMGCR*, *PCSK9* and *LDLR* was quantified using real-time RT-PCR, and the results for WRL-68 and HepG2 cells are shown in Fig 9A and 9B, respectively. The values for mRNA expression are presented as the means ±SEM, n = 3 samples per treatment. Means labeled with (*) are significantly different from the control (0 μM). Western blot analysis was also performed using WRL-68 cell cultures under the same treatment. The results are displayed in Fig 9C.

Considering the decreased transcription of *SREBF2* after luteolin treatment, the mRNA and protein expression of HMGCR were also determined. Real-time RT-PCR showed that 1 μM luteolin reduced the levels of *HMGCR* mRNA by approximately 30% in WRL-68 cells (**Fig 9Ai**) (**S9 Dataset, Table Ai**), and 5 μM luteolin showed a 20% decrease of *HMGCR* mRNA in HepG2 cells (**Fig 9Bi**)(**S9 Dataset, Table Bi**). A similar trend of protein expression was also observed in WRL-68 cells (**Fig 9C**)(**S9 Dataset, Figure C**). Significant reductions in *PCSK9* expression were also observed in cultures treated with >5 μM luteolin (**Fig 9Aii and 9Bii**)(**S9 Dataset, Tables Aii** and **Bii**), whereas no changes were observed in *LDLR* expression (**Fig 9Aiii and 9Biii**)(**S9 Dataset, Tables Aiii** and **Biii**). The C(t) values were estimated as shown in the **S9 Dataset**.

### Cellular cholesterol levels in hepatic cells

**Fig 10:**
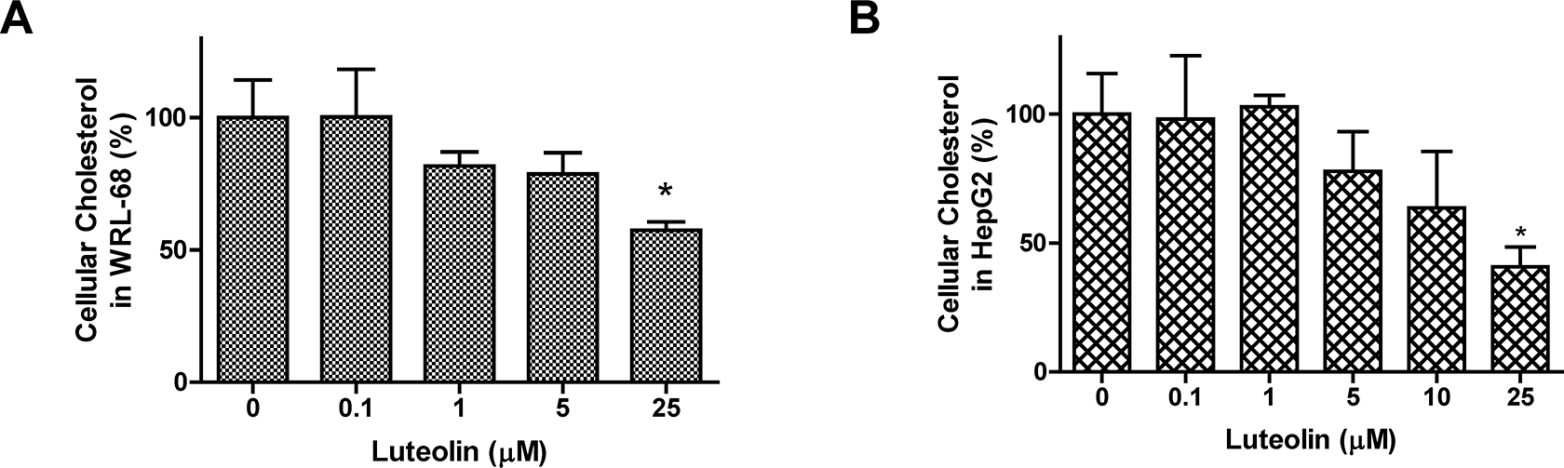
Cellular cholesterol content in luteolin-treated hepatocytes. Hepatic cells were treated with various concentrations of luteolin and cultured for 24 h. The cholesterol content was measured, and the results for WRL-68 and HepG2 cells are shown in Fig 10A and 10B. The values are presented as the means ±SEM, n = 3 samples per treatment. Means labeled with (*) are significantly different from the control (0 μM).

As HMGCR is the key enzyme for cholesterol synthesis, the cellular cholesterol levels were measured. A decreasing trend in the cellular cholesterol levels was observed in WRL-68 cells (**Fig 10A**) or HepG2 cells (**Fig 10B**) under luteolin treatment. The cholesterol levels were significantly (P<0.05) reduced in cells treated with 25 μM luteolin, and the supporting data are provided in the **S10 Dataset, Tables A** and **B**.

### Correlation between AMPK and cellular cholesterol levels

**Fig 11:**
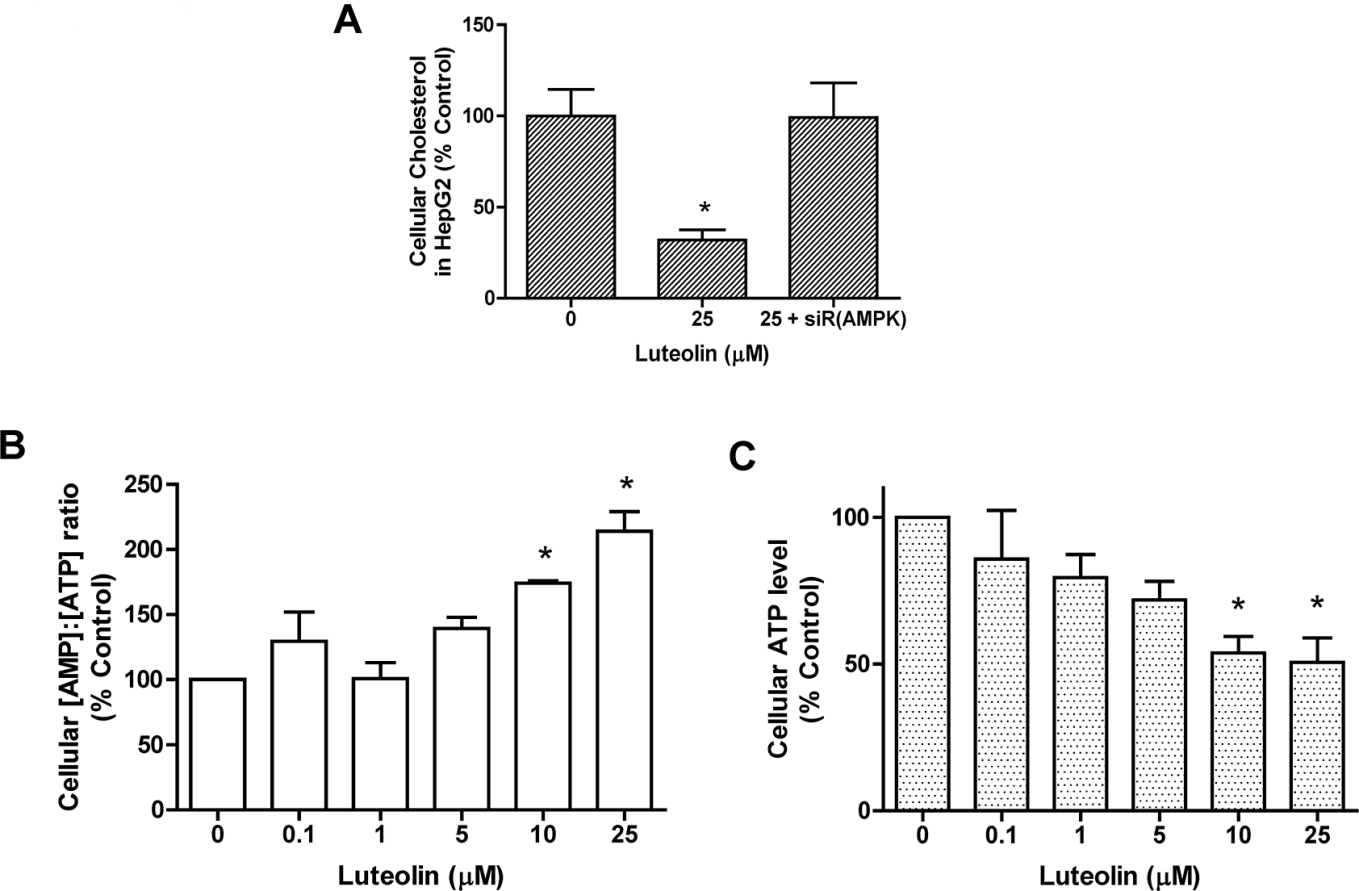
AMPK status and cellular cholesterol content in luteolin-treated cells. HepG2 cells were transfected with AMPKα1 siRNA and treated with 25 μM luteolin for 24 h. The cholesterol content was measured, and the results are shown in Fig 11A. The cellular [AMP]/[ATP] ratios and relative ATP concentrations under luteolin treatments are shown in Fig 11B and 11C, respectively. The values are presented as the means ±SEM, n = 3 samples per treatment. Means labeled with (*) are significantly different from the control (0 μM).

Because luteolin treatment activated AMPK and facilitated SREBP-2 nuclear translocation, the relationship between AMPK and cholesterol was evaluated. Transfecting siRNA directed against AMPK into hepatic cells reversed the reduced cellular cholesterol induced through luteolin (**Fig 11A**)(**S11 Dataset (Table A)**), suggesting an inverse relationship between AMPK and cholesterol. The ratio of the AMP to ATP were also measured. The ratio displayed an increasing trend as the concentration of luteolin administered increased (**Fig 11B**) with the reduction of the ATP concentration (**Fig 11C**)(**Table B** in **S11 Dataset**). These results are consistent with the hypothesis that luteolin increases the AMP concentration and activates AMPK.

## Discussion

In the present study, we demonstrated that luteolin suppresses the expression and perturbs the post-transcriptional processing of SREBP-2 in hepatic cells. Further analysis revealed that the activation of AMPK and deactivation of JNK and PKC could be responsible for these outcomes. As the expression and nuclear translocation of SREBP-2 was reduced, the transcription of the SRE-bearing gene *HMGCR* was downregulated. Although *PCSK9* expression was suppressed, *LDLR* mRNA expression was not affected in this model.

The regulation of *SREBF2* expression is complicated. A feed-forward mechanism has been described for transcriptional control. As SRE sites are also located in the promoter region of *SREBF2*, this transcription factor is also a regulator of its own gene expression [26]. PKB/Akt [24] and hormones, such as insulin and glucagon [26], are also regulators of this gene. JNK2, induced through insulin, is a key mediator for the upregulation of *SREBF1c* in HepG2 cells [25]. Given the similarities between the regulation mechanisms in the same family protein, JNK could also be a regulatory factor in *SREBF2* expression. In the present study, we demonstrated that the SRE-binding activity and pJNK in hepatic cells were reduced through luteolin as two potential mechanisms for the suppression of *SREBF2* mRNA expression. The Akt pathway was unlikely involved, as the Akt-specific inhibitor did not suppress the expression.

PKC might be an upstream regulator of JNK [27, 28], and several PKC isoforms were deactivated through luteolin in the present study. However, the administration of the PKC inhibitor did not induce any significant changes in *SREBF2* mRNA expression. Thus, the hypothesis that PKC controls the activity of JNK could be ruled out in these cells.

Phosphorylation might affect the SRE-interacting activity of SREBP-2. ERK-1/2 phosphorylates this transcription factor and increases binding to SRE [29, 30], whereas the reverse is observed for AMPK [9]. A previous study demonstrated that luteolin activates AMPK in cultured hepatocytes [31]; the results of the present study suggested that flavone also prevented SREBP-2 from post-translational processing and nuclear translocation through the activation of AMPK.

Previous studies have shown that the oral administration of the extracts of *Salix matsudanda* leaves [32] and artichoke [33] reduced plasma cholesterol levels in an animal model. As a major component in these extracts, luteolin has also been demonstrated to be an inhibitor of cholesterol synthesis in primary cultures of rat hepatocytes and HepG2 cells [33, 34]. The results of these studies are consistent with the findings of the present study.

Other natural chemical ingredients isolated from plant foods have also shown plasma cholesterol lowering effects with various actions. Plant stanol esters might achieve this effect through the inhibition of cholesterol absorption. Catechin [35], genistein [36], policosanol [37], and hawthorn extracts [38] have also been reported to prevent cholesterol synthesis through the inhibition of HMGCR. Mulberry anthocyanins reduce the expression of HMGCR through the phosphorylation of AMPK [39]. In contrast, luteolin suppressed SREBP-2 expression and activation in the present study. The reduction of *HMGCR* expression resulted from the compromised SREBP-2 activity.

According to a pharmacokinetic study in rats, an oral dosage of 30 mg luteolin/kg body weight generates a *C*_*max*_ value of 3.12 μM in serum [40]. Similarly, plasma *C*_*max*_ values of 1.16 and 4.31 μM can be obtained after the administration of *p*.*o*. 20 and 100 mg/kg body weight Chrysanthemum morifolium extract [41, 42]. Because luteolin exhibited activity at a concentration as low as 1 μM in the present study, the effective dosage should be physiologically achievable in the form of functional food or dietary supplement.

HMGCR inhibitors are major prescription drugs for alleviating hypercholesterolemia. Increasing the consumption of luteolin-rich vegetables or herbal preparations could be an alternate treatment. In summary, the results of the present study demonstrated that luteolin could attenuate SREBP-2 at the transcriptional and post-translational levels. The downstream genes of SREBP-2, such as *HMGCR*, would also be suppressed.

## Conclusion

In a hepatic cell culture system, luteolin blocked HMGCR by suppressing SREBP-2 transcription and post-translational modification. The results of the present study also illustrated that various phytochemicals isolated from fruits and vegetables might have different effects on *SREBF2* expression.

## Supporting Information

S1 Dataset Experiments for determining mRNA expression in Fig 1. Tables A, B and C are the SREBP-2 expression data in cells treated with various flavonoids and luteolin.
(PDF)

S2 Dataset Immunoblot images for Fig 2. Expression of SREBP-2 in WRL-68 and HepG2 are shown in Figures A and B, respectively.
(PDF)

S3 Dataset Arbitrary light units in Fig 3. The data are listed in Table A.
(PDF)

S4 Dataset MRNA expression in samples treated with kinase inhibitors in Fig 4. The data are listed in Table A.
(PDF)

S5 Dataset Images of PKCs and MAPKs for Fig 5. The images for pPKCs are shown in Figure A, and those for pMAPK are displayed in Figure B.
(PDF)

S6 Dataset Images of AMPK status and nuclear translocation of SREBP-2 for Fig 6. The images acquired from confocal microscopy are shown in Figure A. Western blot results of AMPK and SREBP-2 with Compound C treatment are displayed in Figures B and C, respectively.
(PDF)

S7 Dataset Arbitrary light units in Fig 7. The data are shown in Table A.
(PDF)

S8 Dataset EMSA images for Fig 8. The images are shown in Figure A.
(PDF)

S9 Dataset C(t) values and HMGCR immunoblot in Fig 9. The data for calculating HMGCR, PCSK9, and LDLR expression in WRL-68 cells are shown in Tables Ai, Aii, and Aiii, respectively. Similarly, expression data performed in HepG2 cells are shown in Tables Bi, Bii, and Biii. The images of HMGCR protein blots are displayed in Figure C.
(PDF)

S10 Dataset Absorbance data for measuring protein and cholesterol in Fig 10. Tables A and B are the data of cellular cholesterol in WRL-68 and HepG2 cells, respectively.
(PDF)

S11 Dataset Experiments on AMPK status and cellular cholesterol in Fig 11. The data for calculating cellular cholesterol with AMPK siRNA treatment and AMP to ATP ratio are shown in Tables A and B.
(PDF)
