# Supplementary material for: The Flavone Luteolin Suppresses SREBP-2 Expression and Post-Translational Activation in Hepatic Cells
Source: PLoS One. 2015 Aug 24;10(8):e0135637. doi: 10.1371/journal.pone.0135637 (PMC4547722; doi:10.1371/journal.pone.0135637)
Supplement: S1 Dataset — Tables A, B and C are the SREBP-2 expression data in cells treated with various flavonoids and luteolin. (PDF) [file pone.0135637.s001.pdf]

S1 Dataset. Experiments for mRNA expression in Figure 1.

Table A. Effect of flavonoid on SREBP-2 mRNA expression in WRL-68

Dye	Content	C(t)	GADPH
SREBP-2			
FAM	DMSO	25.19263	16.57002
FAM	DMSO	24.58493	16.05811
FAM	DMSO	26.62566	17.98582
FAM	Luteolin	25.56108	16.51079
FAM	Luteolin	26.19947	17.00865
FAM	Luteolin	26.39807	17.09318
FAM	alpha-naphthoflavone	25.0593	16.83794
FAM	alpha-naphthoflavone	26.02946	16.73566
FAM	alpha-naphthoflavone	25.49691	17.1573
FAM	Baicalein	24.81757	16.22861
FAM	Baicalein	25.49984	16.89592
FAM	Baicalein	25.41132	16.94056
FAM	Chrysin	25.48953	17.08264
FAM	Chrysin	25.61173	16.94534
FAM	Chrysin	26.19125	17.40502
FAM	Flavone	25.95168	17.05856
FAM	Flavone	25.79293	17.4765
FAM	Flavone	25.87611	17.05568
FAM	Genistein	24.92863	16.17824
FAM	Genistein	25.48687	17.39414
FAM	Genistein	24.25462	16.04602

Dye	Content	C(t)	GADPH
SREBP-2			
FAM	DMSO	25.54367	16.68908
FAM	DMSO	25.30554	16.21862
FAM	DMSO	27.64839	19.05478
FAM	Hesperetin	26.42054	17.20727
FAM	Hesperetin	26.65998	17.60353
FAM	Hesperetin	26.24961	16.98111
FAM	Isoliquiritigenin	26.49966	16.97577
FAM	Isoliquiritigenin	26.97661	17.69243
FAM	Isoliquiritigenin	26.58811	17.46455
FAM	Narigenin	25.69193	16.91337
FAM	Narigenin	25.44546	17.00403
FAM	Narigenin	26.57088	17.49743
FAM	Quercetin	26.53421	17.57516
FAM	Quercetin	26.31108	17.20514
FAM	Quercetin	26.04859	17.16572
FAM	Resveretrol	28.09726	18.31664
FAM	Resveretrol	26.25997	17.11879
FAM	Resveretrol	26.44565	17.39326

Table B. Effect of Luteolin on SREBP-2 expression in WRL-68

Dye	Content	C(t)	GADPH
SREBP-2			
FAM	DMSO	25.99541	16.5016
FAM	DMSO	26.37689	16.53882
FAM	DMSO	26.37904	16.55795
FAM	0.1uM Luteolin	26.68173	16.71364
FAM	0.1uM Luteolin	26.87241	16.89095
FAM	0.1uM Luteolin	26.7903	16.89638
FAM	1uM Luteolin	27.33481	16.88421
FAM	1uM Luteolin	26.82653	16.62723
FAM	1uM Luteolin	27.0869	16.78027
FAM	5uM Luteolin	27.86712	16.99517
FAM	5uM Luteolin	27.37634	16.59592
FAM	5uM Luteolin	27.00432	16.39499
FAM	10uM Luteolin	27.88773	17.11728
FAM	10uM Luteolin	27.69572	16.98996
FAM	10uM Luteolin	27.88862	17.18812
FAM	25uM Luteolin	28.33405	17.00082
FAM	25uM Luteolin	28.38232	17.12235
FAM	25uM Luteolin	28.42644	16.98583

Table C. Effect of luteolin on SREBP-2 expression in HepG2

Dye	Content	C(t)	GADPH
SREBP-2			
FAM	DMSO	26.59472	19.02816
FAM	DMSO	26.45532	18.94218
FAM	DMSO	26.67859	19.21393
FAM	0.1uM Luteolin	26.84691	19.44516
FAM	0.1uM Luteolin	26.68072	19.01049
FAM	0.1uM Luteolin	26.9613	19.11782
FAM	1uM Luteolin	27.25425	19.56317
FAM	1uM Luteolin	27.14432	19.37051
FAM	1uM Luteolin	26.86696	18.85275
FAM	5uM Luteolin	27.12911	19.35574
FAM	5uM Luteolin	26.81474	18.84364
FAM	5uM Luteolin	27.58112	19.10752
FAM	10uM Luteolin	28.97942	21.23429
FAM	10uM Luteolin	28.08353	19.52471
FAM	10uM Luteolin	27.66362	19.04733
FAM	25uM Luteolin	28.31525	20.21751
FAM	25uM Luteolin	29.47576	20.96873
FAM	25uM Luteolin	29.47268	21.05141
