# Supplementary material for: The Flavone Luteolin Suppresses SREBP-2 Expression and Post-Translational Activation in Hepatic Cells
Source: PLoS One. 2015 Aug 24;10(8):e0135637. doi: 10.1371/journal.pone.0135637 (PMC4547722; doi:10.1371/journal.pone.0135637)

S2 Dataset. Immunoblot images for Figure 2.

Figure A. Effect of luteolin on SREBP-2 protein expression in WRL-68

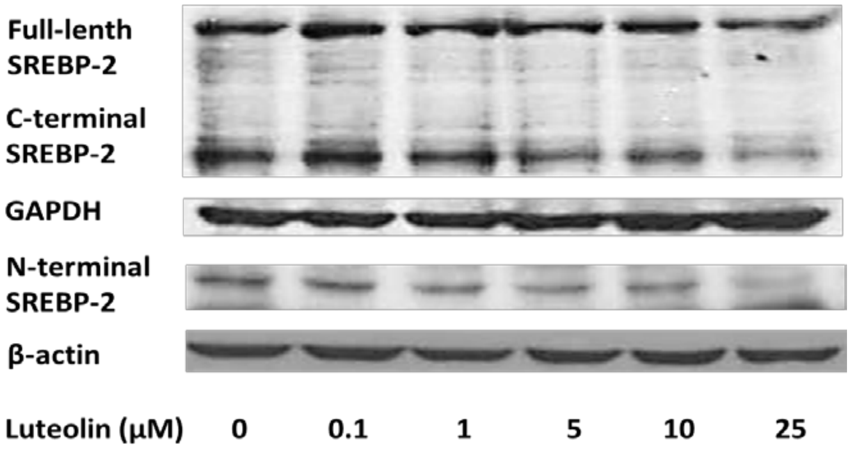

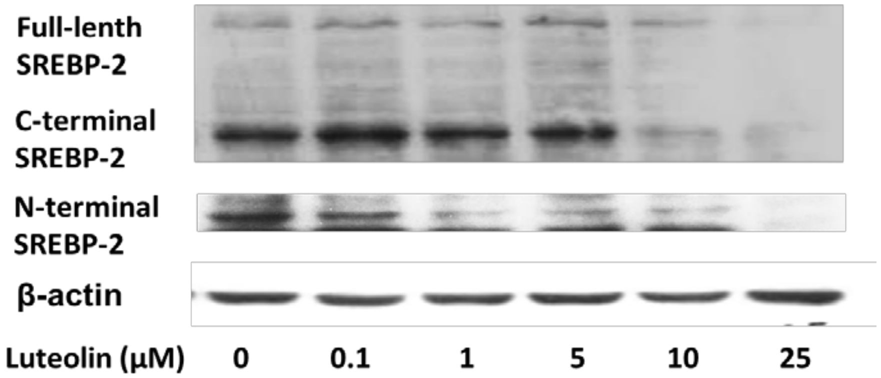

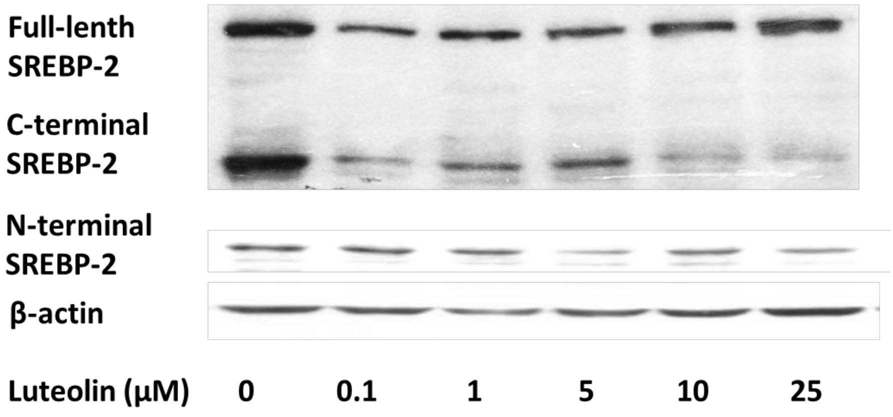

Figure B. Effect of luteolin on SREBP-2 protein expression in HepG2

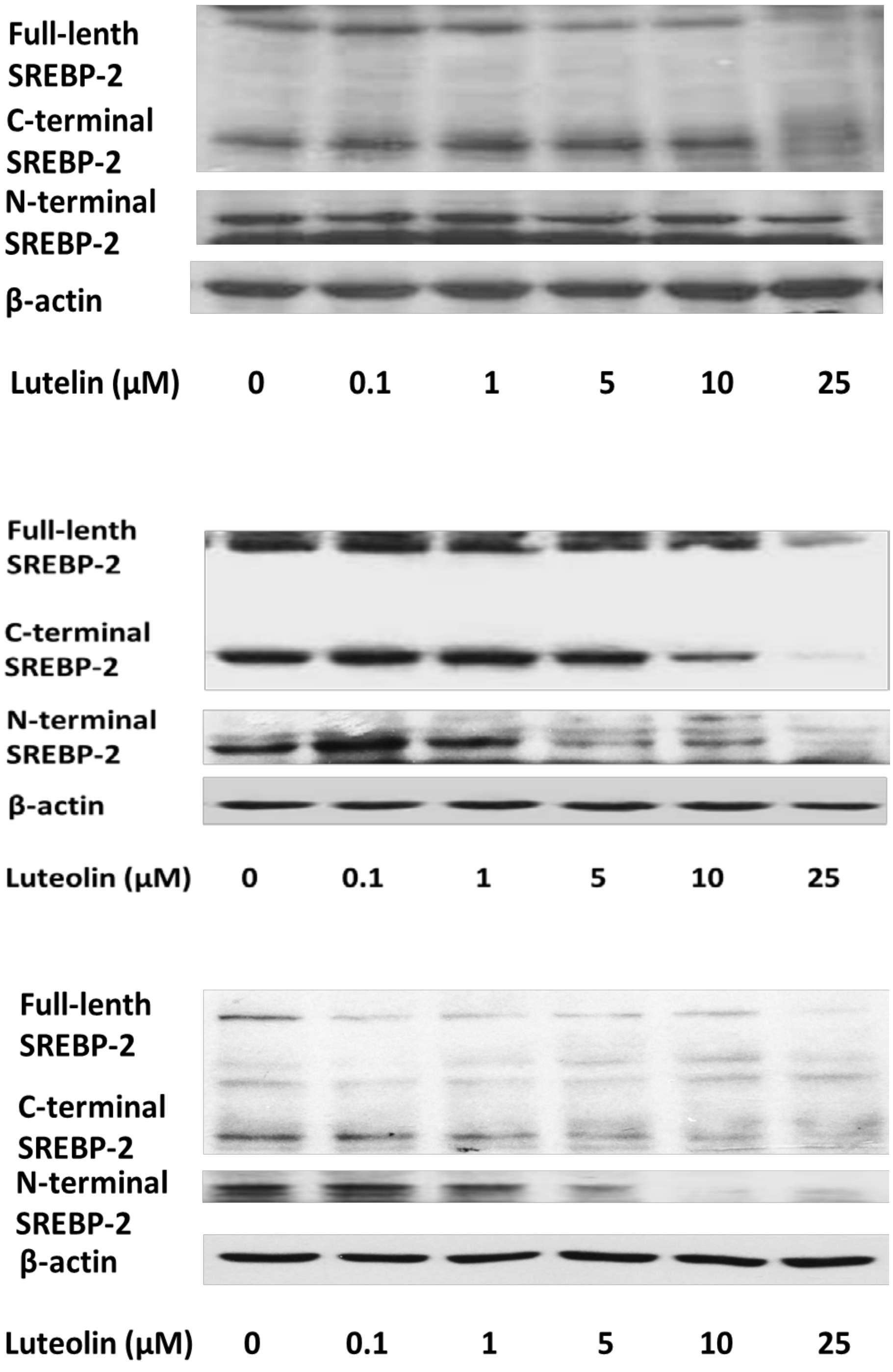

Supplement: S2 Dataset — Expression of SREBP-2 in WRL-68 and HepG2 are shown in Figures A and B, respectively. (PDF) [file pone.0135637.s002.pdf]
