# Supplementary material for: The Flavone Luteolin Suppresses SREBP-2 Expression and Post-Translational Activation in Hepatic Cells
Source: PLoS One. 2015 Aug 24;10(8):e0135637. doi: 10.1371/journal.pone.0135637 (PMC4547722; doi:10.1371/journal.pone.0135637)
Supplement: S3 Dataset — The data are listed in Table A. (PDF) [file pone.0135637.s003.pdf]

S3 Dataset. Arbitrary light units for Figure 3.

Table A.

		firefly (SREBP-2)	Renilla
DMSO	1	602	1040.1765
	2	630.88235	1006.5294
	3	534.88235	1724.5294
	4	474	1106.1765
	5	563	1485.1765
0.1uM Luteolin	1	886	3662.1765
	2	861.88235	1737.1765
	3	809.88235	1809.1765
	4	874	2657.5294
	5	1007	2576.1765
1uM Luteolin	1	831	4117.1765
	2	541.88235	3469.5294
	3	893	2469.5294
	4	805	2664.5294
	5	822	3475.1765
5uM Luteolin	1	761	3764.5294
	2	584	2710.1765
	3	509	1607.1765
	4	957	2421.1765
	5	657	2062.1765
10uM Luteolin	1	1002	3109.1765
	2	681.88235	3025.1765
	3	933	3190.1765
	4	643	2253.1765
	5	649	3395.1765
25uM Luteolin	1	850	3837.5294
	2	505	1916.5294
	3	746.88235	1955.1765
	4	580	3634.5294
	5	813	3095.1765
