# Supplementary material for: The Flavone Luteolin Suppresses SREBP-2 Expression and Post-Translational Activation in Hepatic Cells
Source: PLoS One. 2015 Aug 24;10(8):e0135637. doi: 10.1371/journal.pone.0135637 (PMC4547722; doi:10.1371/journal.pone.0135637)
Supplement: S4 Dataset — The data are listed in Table A. (PDF) [file pone.0135637.s004.pdf]

S4 Dataset. MRNA expression in samples treated with kinase inhibitors in Figure 4.

Table A.

Dye	Content	C(t)	GADPH
SREBP-2			
FAM	DMSO	26.5421	16.63378
FAM	DMSO	26.60255	16.64049
FAM	DMSO	27.10094	17.1351
FAM	BI	27.8289	17.6627
FAM	BI	27.11458	16.98372
FAM	BI	27.41886	17.09923
FAM	HBDDE	27.1293	17.36275
FAM	HBDDE	26.83035	16.69958
FAM	HBDDE	26.9979	16.82335
FAM	LY333531	27.99236	17.6942
FAM	LY333531	27.06503	16.98549
FAM	LY333531	27.35157	16.90317
FAM	H-89	26.98243	17.17479
FAM	H-89	26.36674	16.81832
FAM	H-89	26.55919	17.00784
FAM	pAKT inhibitor	26.5076	16.4606
FAM	pAKT inhibitor	26.32346	16.64665
FAM	pAKT inhibitor	26.56444	16.79019

Dye	Content	C(t)	GADPH
SREBP-2			
FAM	DMSO	23.13139	14.98641
FAM	DMSO	23.46098	15.21282
FAM	DMSO	23.41013	15.34506
FAM	SB203580	22.83655	14.6995
FAM	SB203580	23.10056	15.0781
FAM	SB203580	23.65415	14.94718
FAM	SP600125	24.10046	15.15407
FAM	SP600125	24.48343	15.9539
FAM	SP600125	24.36543	15.17723
FAM	U0126	23.27761	14.78848
FAM	U0126	23.78523	15.36482
FAM	U0126	23.13219	14.45175
FAM	Compound C	23.4819	15.28397

FAM	Compound C	23.61201	15.37146
FAM	Compound C	23.49225	15.28667
