# Supplementary material for: The Flavone Luteolin Suppresses SREBP-2 Expression and Post-Translational Activation in Hepatic Cells
Source: PLoS One. 2015 Aug 24;10(8):e0135637. doi: 10.1371/journal.pone.0135637 (PMC4547722; doi:10.1371/journal.pone.0135637)

S5 Dataset. Images of PKCs and MAPKs in Figure 5.

Figure A. Effect of luteolin on pPKC expression in WRL-68

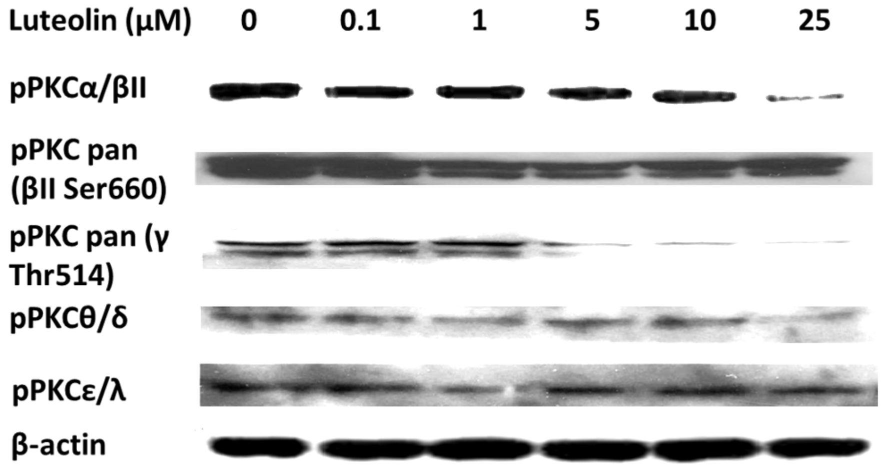

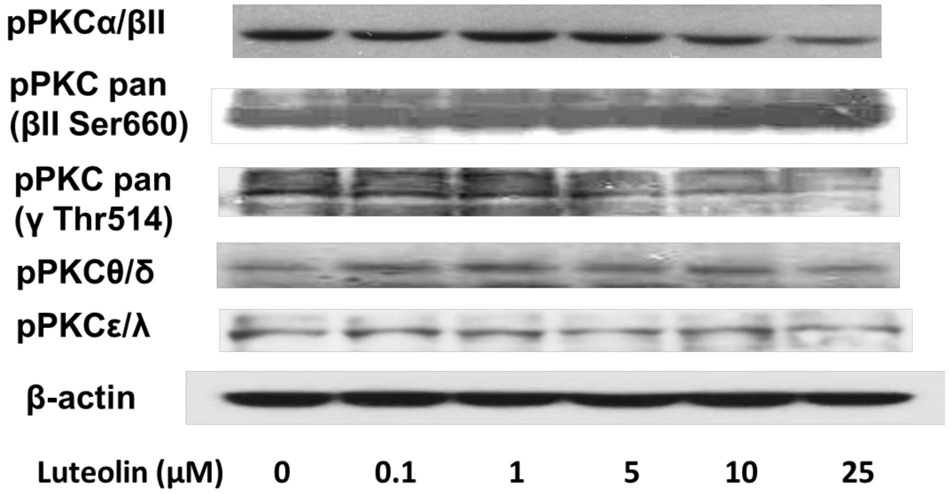

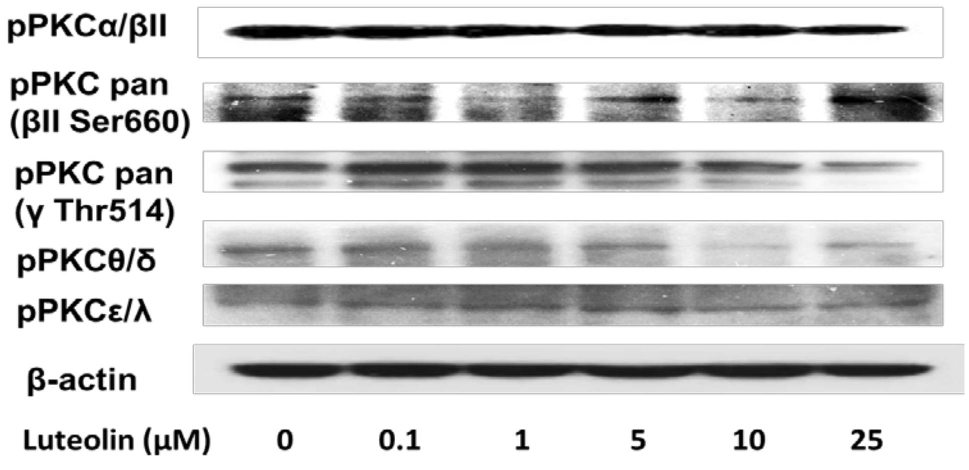

Figure B. Effect of luteolin on pMAPK expression in WRL-68

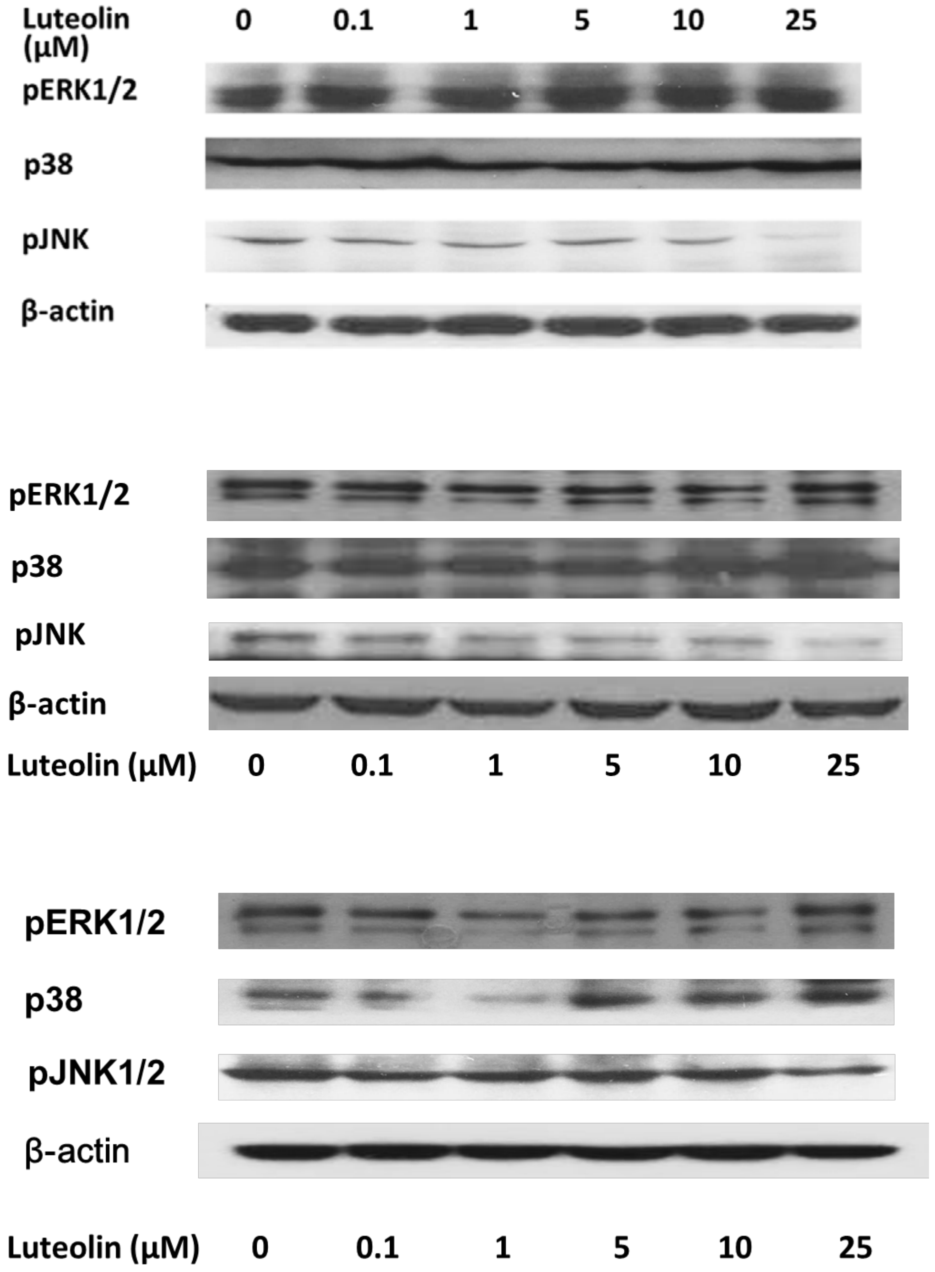

Supplement: S5 Dataset — The images for pPKCs are shown in Figure A, and those for pMAPK are displayed in Figure B. (PDF) [file pone.0135637.s005.pdf]
