# Supplementary material for: The Flavone Luteolin Suppresses SREBP-2 Expression and Post-Translational Activation in Hepatic Cells
Source: PLoS One. 2015 Aug 24;10(8):e0135637. doi: 10.1371/journal.pone.0135637 (PMC4547722; doi:10.1371/journal.pone.0135637)

S6 Dataset. Images of AMPK status and nuclear translocation of SREBP-2 in Figure 6.

Figure A. Nuclear translocation of SREBP-2 in WRL-68

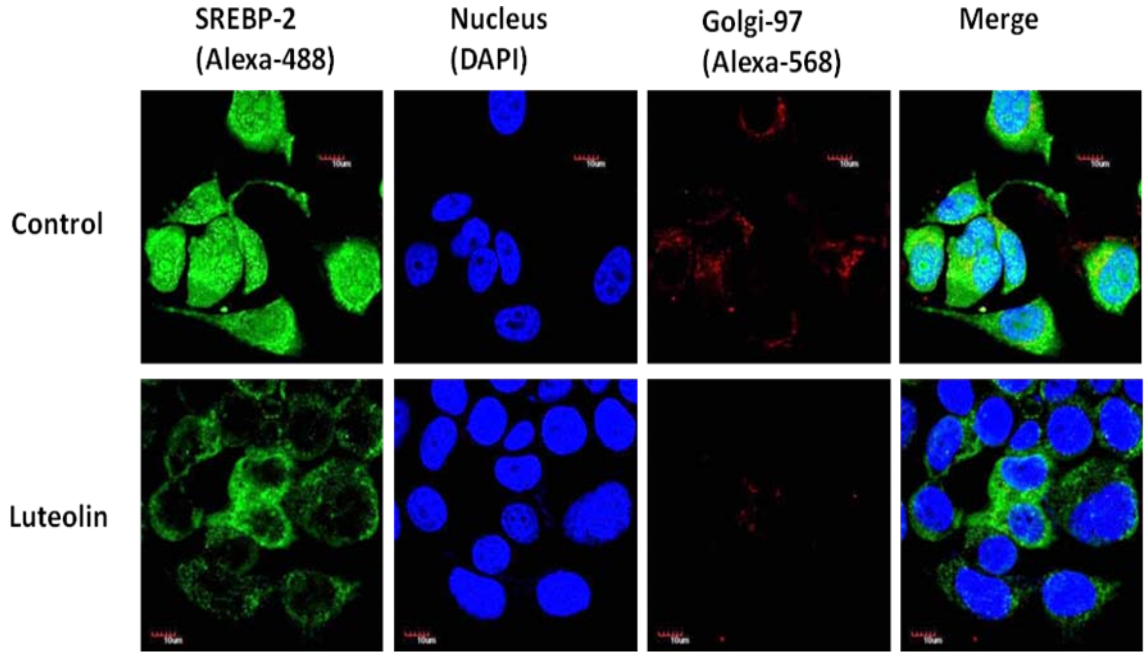

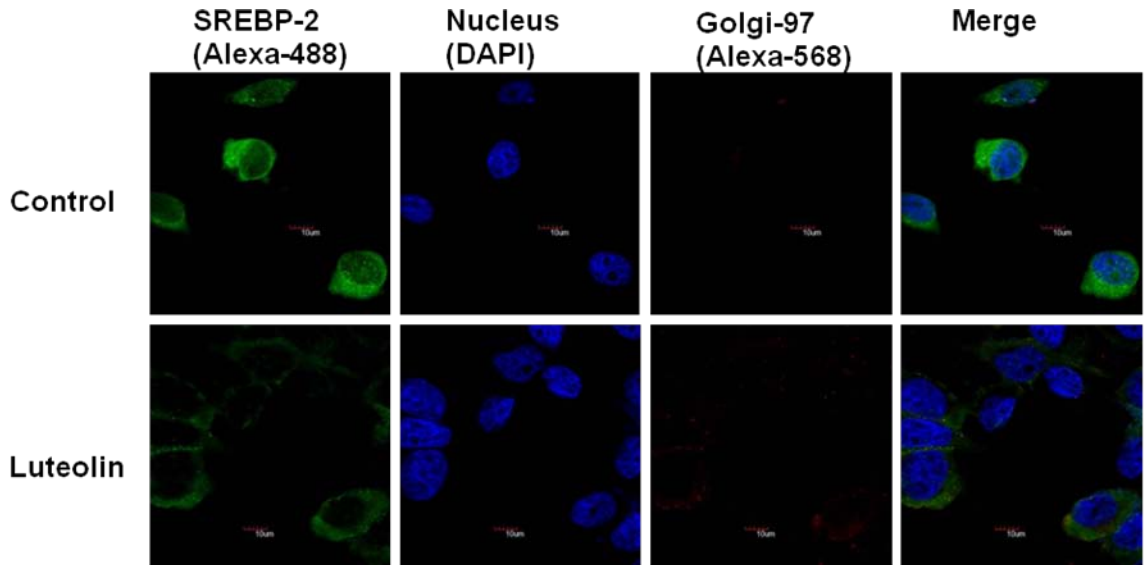

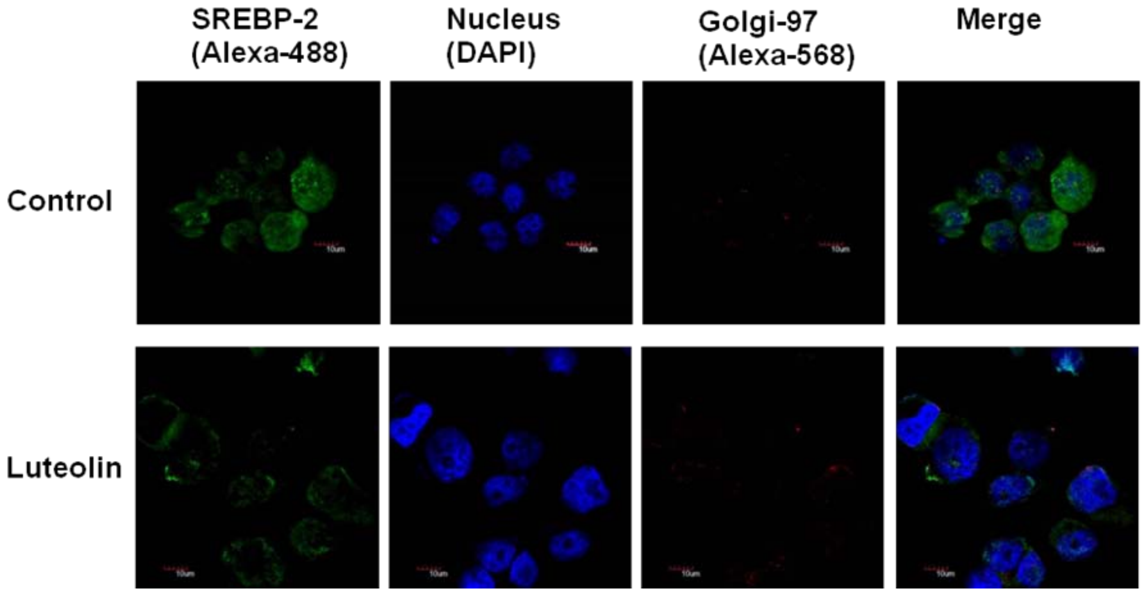

Figure B. Effect of luteolin on AMPK expression in WRL-68

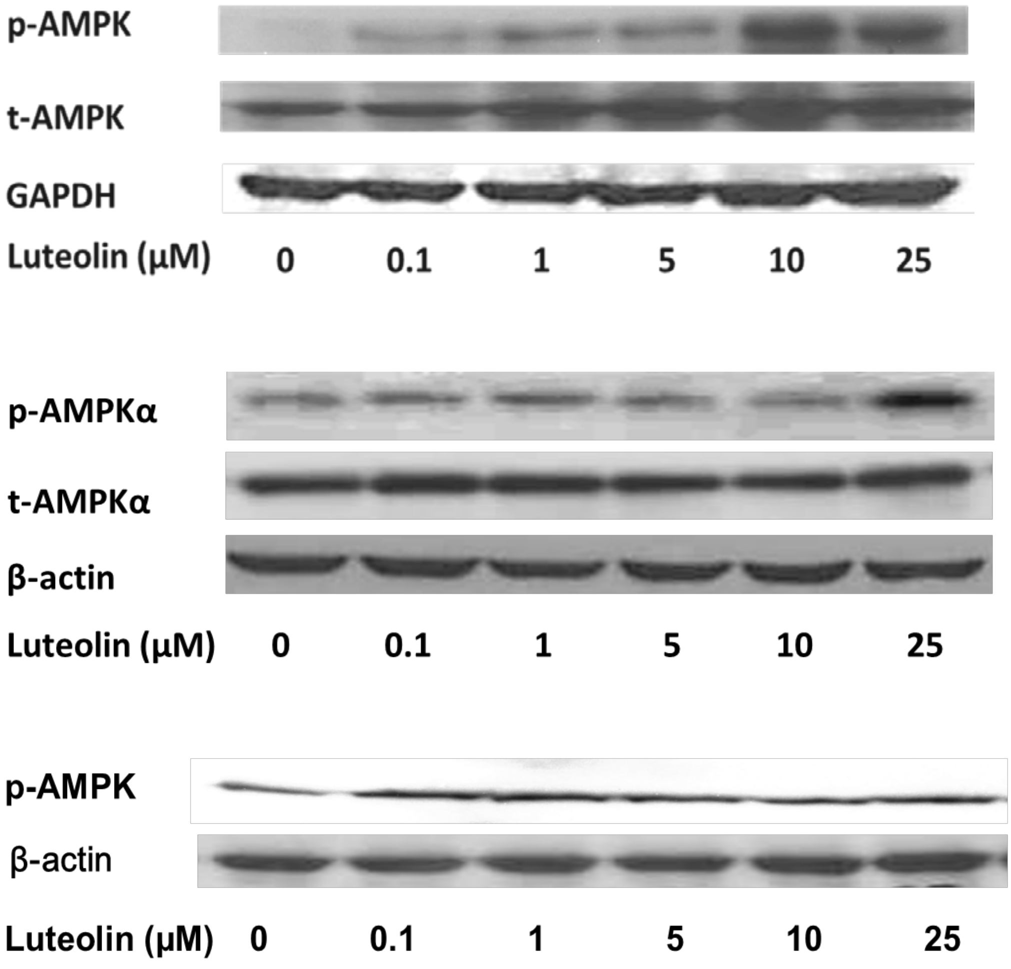

Figure C. Effect of luteolin and AMPK inhibitor Compound C on SREBP-2 expression in WRL-68

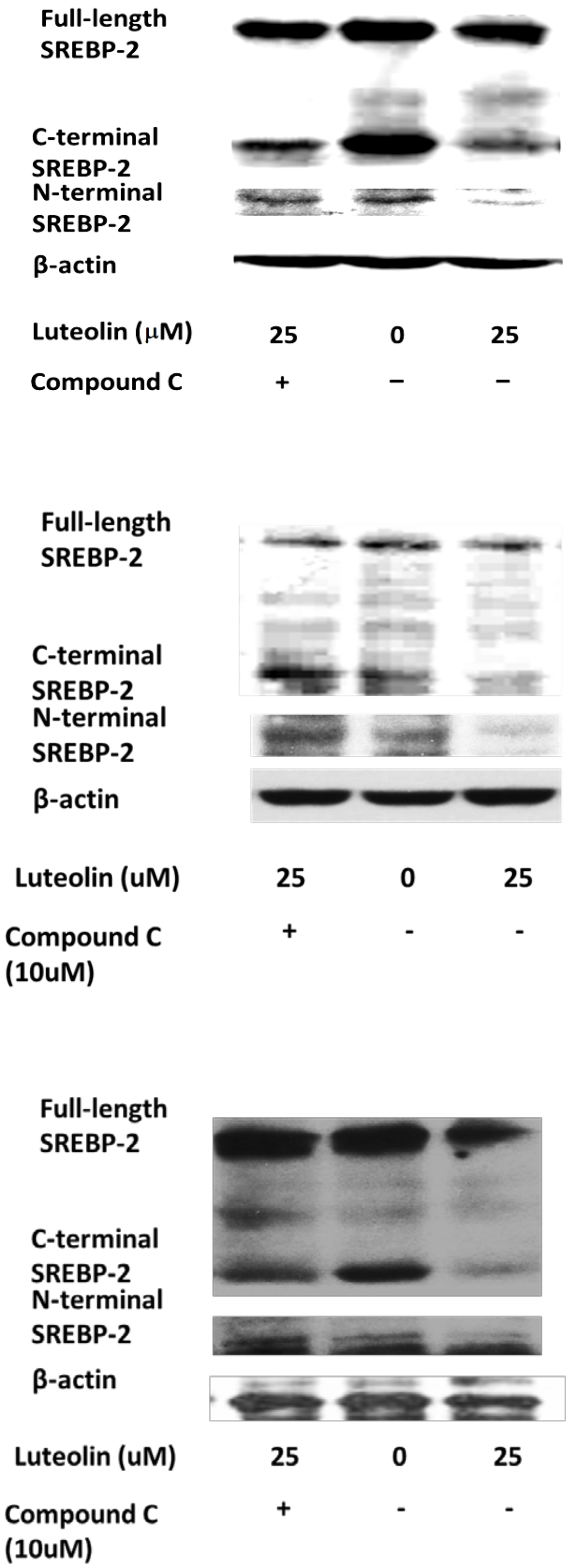

Supplement: S6 Dataset — The images acquired from confocal microscopy are shown in Figure A. Western blot results of AMPK and SREBP-2 with Compound C treatment are displayed in Figures B and C, respectively. (PDF) [file pone.0135637.s006.pdf]
