# Supplementary material for: The Flavone Luteolin Suppresses SREBP-2 Expression and Post-Translational Activation in Hepatic Cells
Source: PLoS One. 2015 Aug 24;10(8):e0135637. doi: 10.1371/journal.pone.0135637 (PMC4547722; doi:10.1371/journal.pone.0135637)
Supplement: S7 Dataset — The data are shown in Table A. (PDF) [file pone.0135637.s007.pdf]

S7 Dataset. Arbitrary light units in Figure 7.

Table A.

		firefly (HMGCR)	renilla
DMSO	1	284.41176	4154
	2	221.41176	3574.8235
	3	334.41176	4962.8235
	4	203.41176	2125
0.1uM Luteolin	1	154.76471	2987
	2	129.76471	2748.8235
	3	109.41176	3672
	4	245.41176	6865
1uM Luteolin	1	126.76471	5719.8235
	2	127.41176	7263.8235
	3	127.41176	6484
5uM Luteolin	1	82.411765	4563.8235
	2	123.76471	7390
	3	91.411765	4593
10uM Luteolin	1	43.411765	4050.8235
	2	32.764706	4146.8235
	3	91.411765	4593
	4	33.411765	4135.8235
25uM Luteolin	1	28.764706	3719.8235
	2	22.411765	5341
	3	26.411765	4736.8235
