# Supplementary figures and images for: The Flavone Luteolin Suppresses SREBP-2 Expression and Post-Translational Activation in Hepatic Cells
Source: PLoS One. 2015 Aug 24;10(8):e0135637. doi: 10.1371/journal.pone.0135637 (PMC4547722; doi:10.1371/journal.pone.0135637)

## S8 Dataset. EMSA images in Figure 8.

Figure A.

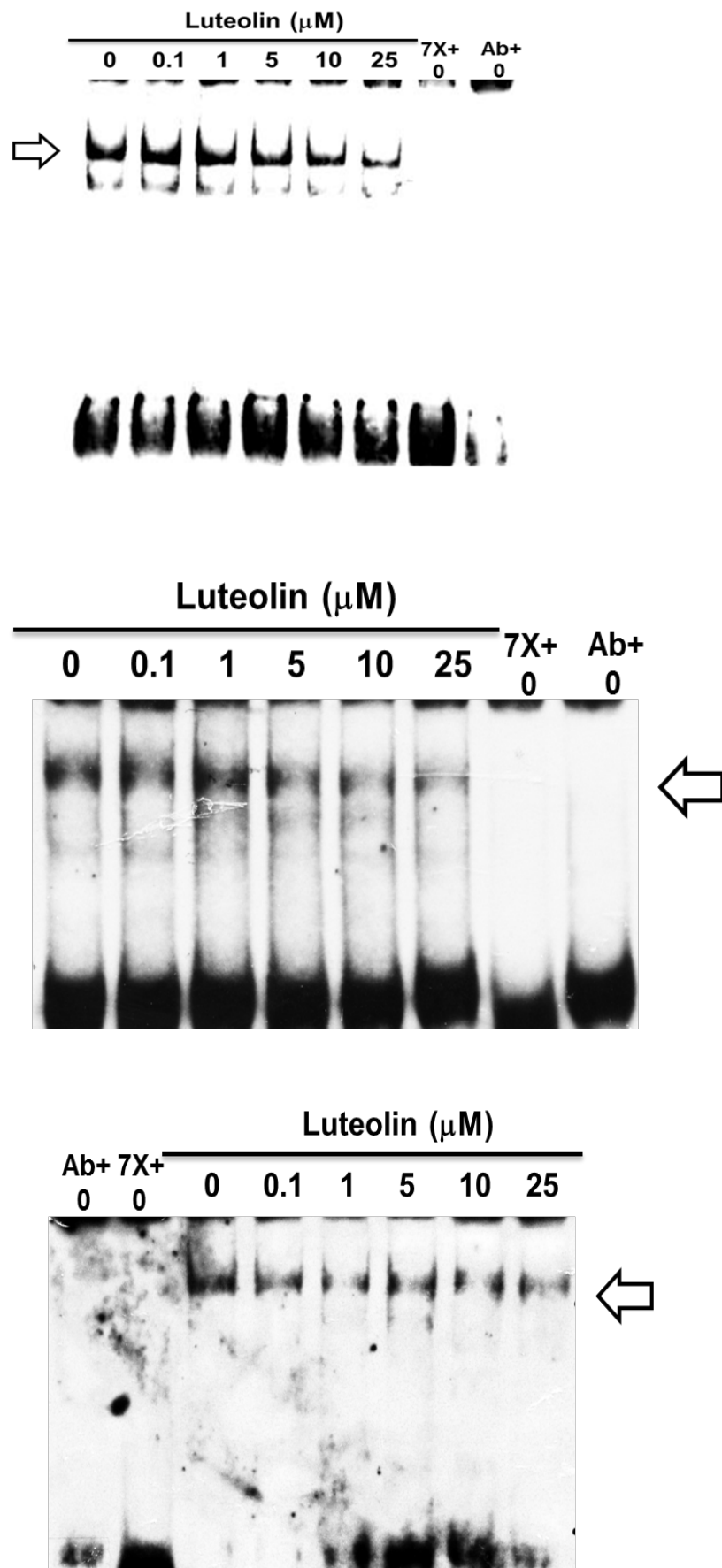

Supplement: S8 Dataset — The images are shown in Figure A. (PDF) [file pone.0135637.s008.pdf]
