# Supplementary material for: The Flavone Luteolin Suppresses SREBP-2 Expression and Post-Translational Activation in Hepatic Cells
Source: PLoS One. 2015 Aug 24;10(8):e0135637. doi: 10.1371/journal.pone.0135637 (PMC4547722; doi:10.1371/journal.pone.0135637)

S9 Dataset. Experiments on mRNA Expression in Figure 9.

Table Ai. Effect of luteolin on HMGCR expression in WRL-68

Dye	Content	C(t)	GADPH
HMGCR			
FAM	DMSO	24.58942	18.43502
FAM	DMSO	24.40609	17.96993
FAM	DMSO	24.3262	18.13807
FAM	0.1uM Luteolin	24.95616	18.58042
FAM	0.1uM Luteolin	24.43231	18.14989
FAM	0.1uM Luteolin	24.94299	18.37944
FAM	1uM Luteolin	25.47574	18.4331
FAM	1uM Luteolin	24.82911	18.16461
FAM	1uM Luteolin	24.91658	18.51049
FAM	5uM Luteolin	25.86914	18.33366
FAM	5uM Luteolin	24.73366	18.15283
FAM	5uM Luteolin	25.33522	18.12295
FAM	10uM Luteolin	25.56937	18.50446
FAM	10uM Luteolin	25.55986	18.35249
FAM	10uM Luteolin	25.30577	18.07431
FAM	25uM Luteolin	26.69709	18.52396
FAM	25uM Luteolin	26.48577	18.43606
FAM	25uM Luteolin	26.54617	18.778

Table Aii. Effect of luteolin on PCSK9 expression in WRL-68

Dye	Content	C(t)	GADPH
PCSK9			
FAM	DMSO	22.17317	12.65241
FAM	DMSO	23.32314	13.77002
FAM	DMSO	22.2411	12.55935
FAM	0.1uM Luteolin	22.9145	13.2361
FAM	0.1uM Luteolin	23.03209	13.14285
FAM	0.1uM Luteolin	22.81734	13.55543
FAM	1uM Luteolin	22.88755	13.10037
FAM	1uM Luteolin	23.16284	12.97639
FAM	1uM Luteolin	23.54178	13.12185
FAM	5uM Luteolin	23.57263	13.14967

FAM	5uM Luteolin	23.53804	13.35923
FAM	5uM Luteolin	23.36374	13.18041
FAM	10uM Luteolin	24.15307	14.12633
FAM	10uM Luteolin	23.78887	13.52404
FAM	10uM Luteolin	23.9683	13.62398
FAM	25uM Luteolin	25.42448	15.22054
FAM	25uM Luteolin	25.24813	14.84998
FAM	25uM Luteolin	25.49118	15.1728

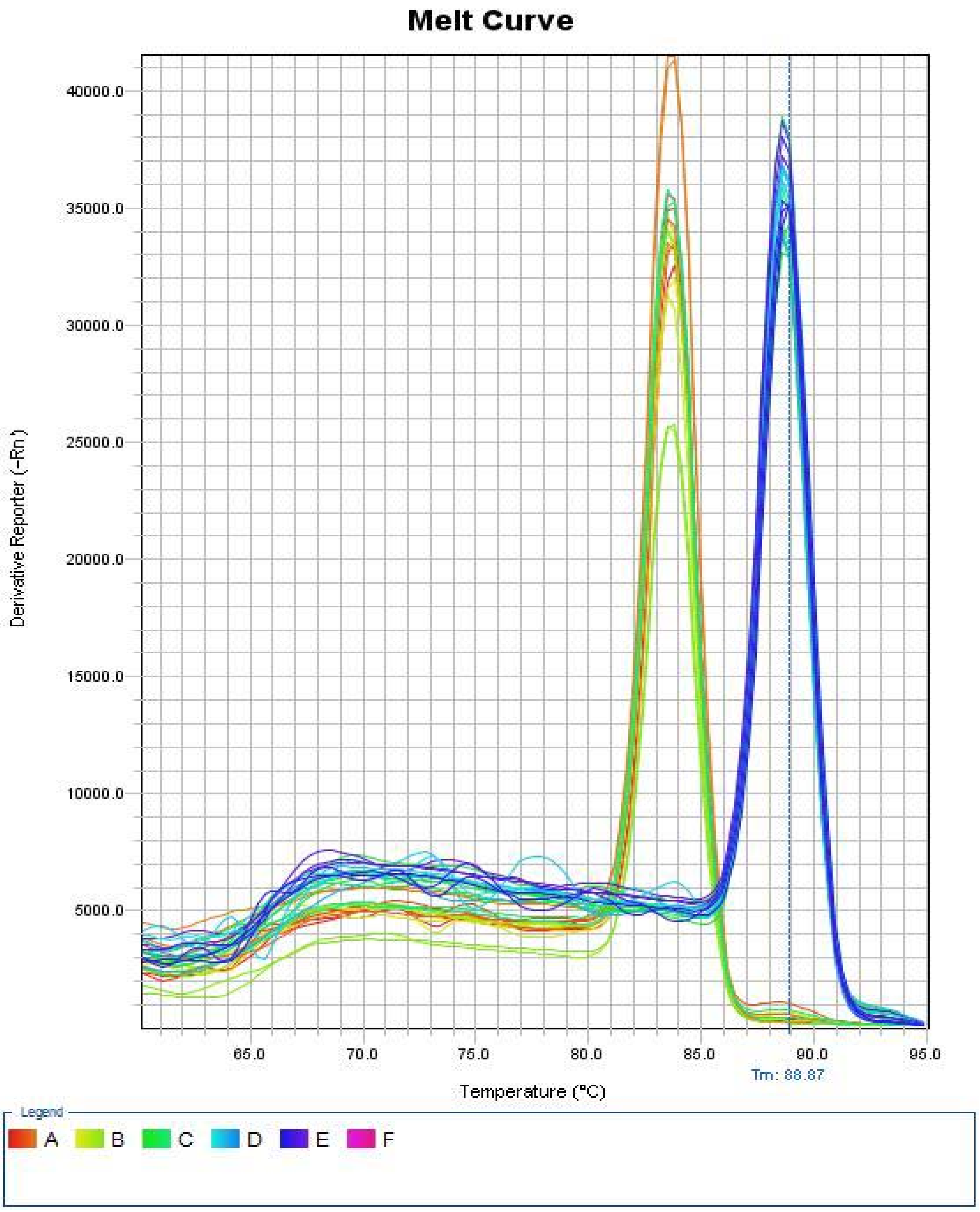

Table Aiii. Effect of luteolin on LDLR expression in WRL-68

Dye	Content	C(t)	GADPH
LDLR			
FAM	DMSO	27.91017	18.20043
FAM	DMSO	28.4031	18.64404
FAM	DMSO	28.46386	18.42223
FAM	0.1uM Luteolin	27.78505	18.13291
FAM	0.1uM Luteolin	27.49096	17.79722
FAM	0.1uM Luteolin	27.75152	17.96507
FAM	1uM Luteolin	28.62938	18.25868
FAM	1uM Luteolin	29.10219	18.91041
FAM	1uM Luteolin	28.97728	18.58455
FAM	5uM Luteolin	28.50045	18.35008
FAM	5uM Luteolin	28.84067	18.54067
FAM	5uM Luteolin	28.79609	18.44538
FAM	10uM Luteolin	29.18331	18.97722
FAM	10uM Luteolin	28.95917	18.70349
FAM	10uM Luteolin	29.2524	18.84035
FAM	25uM Luteolin	28.9641	19.32469
FAM	25uM Luteolin	28.53982	18.90956
FAM	25uM Luteolin	28.90437	19.11713

Table Bi. Effect of luteolin on HMGCR in HepG2

Dye	Content	C(t)	GADPH
HMGCR			
FAM	DMSO	25.60993	19.16998
FAM	DMSO	26.46027	20.21478
FAM	DMSO	25.39909	19.00295
FAM	0.1uM Luteolin	26.06255	19.77556
FAM	0.1uM Luteolin	26.03781	19.6962
FAM	0.1uM Luteolin	26.16613	19.79111
FAM	1uM Luteolin	25.73788	19.40932
FAM	1uM Luteolin	26.28748	19.80731
FAM	1uM Luteolin	26.0223	19.67801
FAM	5uM Luteolin	26.25183	19.64976
FAM	5uM Luteolin	26.36216	19.59191
FAM	5uM Luteolin	26.07876	19.53993

FAM	10uM Luteolin	26.25338	19.53205
FAM	10uM Luteolin	26.8226	20.28639
FAM	10uM Luteolin	26.55061	19.58265
FAM	25uM Luteolin	28.46865	21.09488
FAM	25uM Luteolin	28.9019	21.52405
FAM	25uM Luteolin	28.57964	21.22915

Table Bii. Effect of luteolin on PCSK9 in HepG2

Dye	Content	C(t)	GADPH
PCSK9			
FAM	DMSO	22.32314	12.65241
FAM	DMSO	23.67317	13.77002
FAM	DMSO	21.6411	12.55935
FAM	0.1uM Luteolin	22.9145	13.2361
FAM	0.1uM Luteolin	22.83209	13.14285
FAM	0.1uM Luteolin	23.31734	13.55543
FAM	1uM Luteolin	22.88755	13.10037
FAM	1uM Luteolin	23.16284	12.97639
FAM	1uM Luteolin	23.54178	13.12185
FAM	5uM Luteolin	23.57263	13.14967
FAM	5uM Luteolin	23.53804	13.35923
FAM	5uM Luteolin	23.36374	13.18041
FAM	10uM Luteolin	24.15307	14.12633
FAM	10uM Luteolin	23.78887	13.52404
FAM	10uM Luteolin	23.9683	13.62398
FAM	25uM Luteolin	25.42448	15.22054
FAM	25uM Luteolin	25.24813	14.84998
FAM	25uM Luteolin	25.49118	15.1728

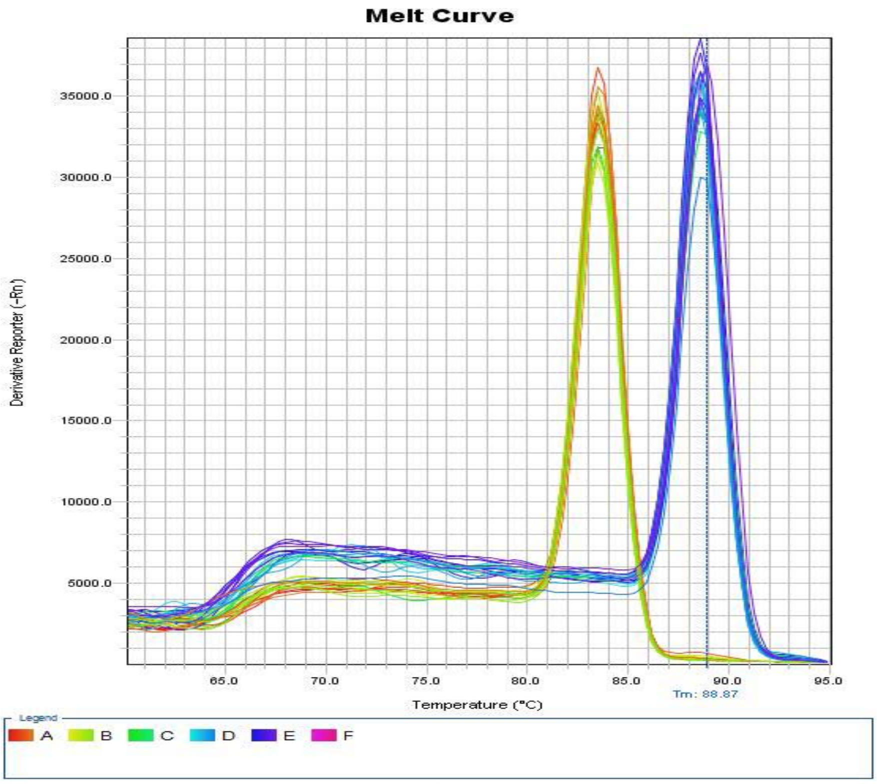

Table Biii. Effect of luteolin on LDLR expression in HepG2

Dye	Content	C(t)	GADPH
LDLR			
FAM	DMSO	27.62154	19.55793
FAM	DMSO	27.37638	19.32828
FAM	DMSO	27.68689	19.45799
FAM	0.1uM Luteolin	28.15743	19.8436
FAM	0.1uM Luteolin	28.02131	19.65837
FAM	0.1uM Luteolin	27.70275	19.48459
FAM	1uM Luteolin	28.05857	19.66932
FAM	1uM Luteolin	27.47332	19.25667
FAM	1uM Luteolin	28.03046	19.58992
FAM	5uM Luteolin	28.11374	19.62802
FAM	5uM Luteolin	27.40342	19.10932
FAM	5uM Luteolin	28.4918	19.74939
FAM	10uM Luteolin	28.91778	20.50377
FAM	10uM Luteolin	28.27016	19.94657
FAM	10uM Luteolin	28.11263	20.00469
FAM	25uM Luteolin	29.66792	21.65942
FAM	25uM Luteolin	29.75455	21.45296
FAM	25uM Luteolin	29.95363	21.77366

Figure C. Effect of luteolin on HMGCR protein expression in WRL-68

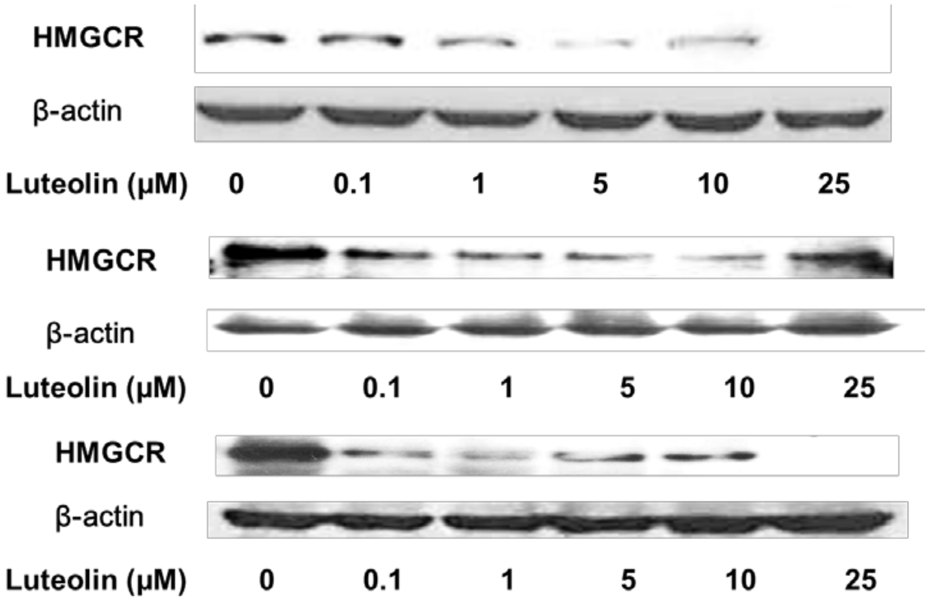

Supplement: S9 Dataset — The data for calculating HMGCR, PCSK9, and LDLR expression in WRL-68 cells are shown in Tables Ai, Aii, and Aiii, respectively. Similarly, expression data performed in HepG2 cells are shown in Tables Bi, Bii, and Biii. The images of HMGCR protein blots are displayed in Figure C. (PDF) [file pone.0135637.s009.pdf]
