# Supplementary material for: The Flavone Luteolin Suppresses SREBP-2 Expression and Post-Translational Activation in Hepatic Cells
Source: PLoS One. 2015 Aug 24;10(8):e0135637. doi: 10.1371/journal.pone.0135637 (PMC4547722; doi:10.1371/journal.pone.0135637)
Supplement: S10 Dataset — Tables A and B are the data of cellular cholesterol in WRL-68 and HepG2 cells, respectively. (PDF) [file pone.0135637.s010.pdf]

S10 Dataset. Absorbance for measuring protein and cholesterol in Figure 10.

Table A. Effect of luteolin on cellular cholesterol in WRL-68

Group		BCA Assay	OD500
Control	1	1.456231	0.13605
	2	1.867518	0.11435
	3	1.161874	0.07405
0.1uM Luteolin	1	1.592807	0.14705
	2	1.456422	0.06965
	3	1.023465	0.08055
1uM Luteolin	1	1.89028	0.12175
	2	1.980253	0.12335
	3	1.62151	0.08385
5uM Luteolin	1	1.342417	0.08825
	2	1.024322	0.04715
	3	1.96213	0.11785
10uM Luteolin	1	1.432138	0.07855
	2	1.000977	0.09525
	3	1.381322	0.08705
25uM Luteolin	1	1.132468	0.05245
	2	1.618165	0.06285
	3	2.000977	0.08095

OD500 for cholesterol standard (200mg/dL): 0.2669

Table B. Effect of luteolin on cellular cholesterol in HepG2

Group		BCA Assay	OD500
Control	1	2.228604	0.0537
	2	2.000977	0.0816
	3	2.046502	0.0597
0.1uM Luteolin	1	2.602158	0.1083
	2	3.870635	0.0611
	3	2.306243	0.0803
1uM Luteolin	1	3.571603	0.1244
	2	2.867518	0.0912
	3	2.456231	0.0739
5uM Luteolin	1	3.046502	0.0875
	2	3.296892	0.0488
	3	3.296892	0.098
10uM Luteolin	1	3.862124	0.0447
	2	3.073632	0.103
	3	3.573161	0.0529
25uM Luteolin	1	2.424354	0.023
	2	1.456231	0.0166
	3	3.323425	0.0581

OD500 for cholesterol standard (200mg/dL): 0.27575
