# Supplementary material for: The Flavone Luteolin Suppresses SREBP-2 Expression and Post-Translational Activation in Hepatic Cells
Source: PLoS One. 2015 Aug 24;10(8):e0135637. doi: 10.1371/journal.pone.0135637 (PMC4547722; doi:10.1371/journal.pone.0135637)
Supplement: S11 Dataset — The data for calculating cellular cholesterol with AMPK siRNA treatment and AMP to ATP ratio are shown in Tables A and B. (PDF) [file pone.0135637.s011.pdf]

S11 Dataset. Experiment on AMPK status and cellular cholesterol in Figure 11.

Table A. Effect of luteolin and AMPK siRNA in cellular cholesterol in HepG2

Group		BCA Assay	OD500
Control	1	0.119695	2.0845
	2	0.091406	2.0398
	3	0.066652	1.9267
25uM	1	0.338939	1.8689
	2	0.185115	1.2284
	3	0.190419	1.8664
25uM+AMPK siRNA	1	0.102129	1.4624
	2	0.048971	1.4194
	3	0.063116	1.5638

OD500 for cholesterol standard (200mg/dL): 0.27575

Table B. Effect of luteolin on cellular [AMP]:[ATP] ratio in WRL-68

Standard curve for 1st-2nd Trial	
Concentration of ATP	RLU
100uM	4213
50uM	2147
25uM	1099
12.5uM	449
0uM	0

1st Trial:

Luteolin (uM)	0	0.1	1	5	10	25
AMP+ADP+ATP	9808	8082	9887	9590	8655	8439
ADP+ATP	9118	7561	9240	8614	7697	7424
ATP	7452	6099	7777	6981	6054	5729
BCA Assay	6.064977949	4.178049	6.907901	6.784134	7.579777	6.996306

2nd Trial:

Luteolin (uM)	0	0.1	1	5	10	25
AMP+ADP+ATP	5167	6161	5871	7690	9515	3737
ADP+ATP	4431	5533	5199	6089	8386	3273
ATP	3065	4626	3919	4869	6642	2789
BCA Assay	6.448196	4.738906	5.617191	10.76851	10.51111	4.576066

3rd Trial:

Standard curve for 3rd Trial	
Concentration of ATP	RLU
100uM	4183
50uM	2208
25uM	466
0uM	0

Luteolin (uM)	0	0.1	1	5	10	25
AMP+ADP+ATP	8263	6828	8095	8688	9414	8242
ADP+ATP	7533	5960	7486	7673	8145	6942
ATP	5585	3907	5350	5378	5562	4762
BCA Assay	7.450009245	7.765441	8.710443	10.34088	15.5737	16.46435
